# Whisker pad stimulation with different frequencies reveals non-uniform modulation of functional MRI signal across sensory systems in awake rats

**DOI:** 10.1093/cercor/bhaf194

**Published:** 2025-07-23

**Authors:** Jaakko Paasonen, Juha S Valjakka, Raimo A Salo, Ekaterina Paasonen, Heikki Tanila, Shalom Michaeli, Silvia Mangia, Olli Gröhn

**Affiliations:** A. I. Virtanen Institute for Molecular Sciences, University of Eastern Finland, Neulaniementie 2, FI-70211, Kuopio, Finland; A. I. Virtanen Institute for Molecular Sciences, University of Eastern Finland, Neulaniementie 2, FI-70211, Kuopio, Finland; Center for Magnetic Resonance Research, University of Minnesota, 2021 Sixth Street SE, Minneapolis, MN 55455, United States; A. I. Virtanen Institute for Molecular Sciences, University of Eastern Finland, Neulaniementie 2, FI-70211, Kuopio, Finland; A. I. Virtanen Institute for Molecular Sciences, University of Eastern Finland, Neulaniementie 2, FI-70211, Kuopio, Finland; NeuroCenter, Kuopio University Hospital, Puijonlaaksontie 2, FI-70210, Kuopio, Finland; A. I. Virtanen Institute for Molecular Sciences, University of Eastern Finland, Neulaniementie 2, FI-70211, Kuopio, Finland; Center for Magnetic Resonance Research, University of Minnesota, 2021 Sixth Street SE, Minneapolis, MN 55455, United States; Center for Magnetic Resonance Research, University of Minnesota, 2021 Sixth Street SE, Minneapolis, MN 55455, United States; A. I. Virtanen Institute for Molecular Sciences, University of Eastern Finland, Neulaniementie 2, FI-70211, Kuopio, Finland

**Keywords:** awake, functional magnetic resonance imaging, rat, stimulation, zero echo time

## Abstract

Primary sensory systems are traditionally considered separate units, but emerging evidence highlights notable interactions between them. Using a quiet and motion-tolerant zero-echo time functional magnetic resonance imaging technique, we examined brain-wide cross-sensory responses to whisker pad stimulation in awake and anesthetized rats. Our results indicate that whisker pad stimulation activated not only the whisker-mediated tactile system, but also auditory, visual, high-order, and cerebellar regions, demonstrating brain-wide cross-sensory and associative activity. Based on response characteristics, non-core regions responded to stimulation in a markedly different way compared to the primary sensory system, likely reflecting distinct encoding modes among primary sensory, cross-sensory, and integrative processing. Lastly, while low-order sensory activity was detectable under anesthesia, high-order processing and the complex differences between primary, cross-sensory, and associative systems were evident only in the awake state. This study reveals novel aspects of the cross-sensory interplay of the whisker-mediated tactile system and underscores the challenges of observing these phenomena in anesthetized rats.

## Introduction

The perception by the senses of surrounding events and objects is one of the most important tasks undertaken by the brain (Petersen 2007; Stein and Stanford 2008). The core structures of primary sensory circuits have been extensively studied using electrophysiological, histological, and neuroimaging techniques. However, while sensory research often focuses on individual sensory systems, compelling evidence suggests that sensory circuits form an integrated system at multiple levels of the ascending pathways (Ghazanfar and Schroeder 2006a; Driver and Noesselt 2008; Stein and Stanford 2008; Yau et al. 2015a). For example, the whisker-mediated tactile system in rodents connects mechanoreceptors around whisker shafts to the barrel region of the primary somatosensory cortex (S1bf) via brainstem and thalamic nuclei (Petersen 2007; Adibi 2019). This system, however, also projects to various, likely multisensory regions, such as inferior colliculus (IC) (Coleman and Clerici 1987), superior colliculus (SC) (Veinante et al. 2000; Ebbesen et al. 2018; Adibi 2019), medial geniculate nuclei (MGN) (Veinante et al. 2000), zona incerta (ZI) (Adibi 2019), posterior parietal cortex (PPC) (Mohan et al. 2018), and secondary somatosensory cortex (S2) (Bale and Maravall 2018; Adibi 2019). Due to the recent emphasis on cross- and multisensory perspective (Driver and Noesselt 2008) and findings, such that an auditory stimulus can induce or modulate neural activity in visual cortex (Vis) or vice versa (Driver and Noesselt 2008; Yau et al. 2015b; Petro et al. 2017), it does seem that many thalamic nuclei and parts of primary cortices should be considered multisensory rather than specific to a single sense (Ghazanfar and Schroeder 2006b; Driver and Noesselt 2008; Yau et al. 2015b).

Studies of brain-wide cross-sensory interactions are challenging for electrophysiological techniques due to their limited spatial coverage, but ideal for modern neuroimaging methods. Indeed, several functional magnetic resonance imaging (fMRI) studies in humans (Raij et al. 2010; Meyer et al. 2011; Man et al. 2015; Smith and Goodale 2015; Gu et al. 2020) and non-human primates (Kayser et al. 2007; Guipponi et al. 2015) have explored signal changes in non-core sensory regions in response to unisensory stimuli, addressing fundamental questions like which non-core regions are activated by inputs to core sensory circuits. However, more complex questions, such as how non-core circuits respond to varying inputs to the core sensory circuit, have remained unexplored (Dinh et al. 2021, 2024). Moreover, previous work has primarily focused on the cortical auditory–visual interactions, with only few studies in humans (Man et al. 2015; Smith and Goodale 2015), non-human primates (Guipponi et al. 2015), and anesthetized mice (Dinh et al. 2024) examining cross-sensory responses to tactile stimuli. No whisker-related fMRI studies (Yangt et al. 1996; Lu et al. 2003, 2005; Sachdev et al. 2003; De Celis Alonso et al. 2008; Sanganahalli et al. 2008, 2022; de Celis Alonso et al. 2012; Devonshire et al. 2012; Yu et al. 2012; Martin et al. 2013a) have reported cross-sensory activity in rats. Notably, most of the rat studies were conducted under anesthesia, which affects the organization of brain (Paasonen et al. 2018) and potentially also cross-sensory processing (Dinh et al. 2024). Lastly, the loud noise inherent to traditional fMRI sequences poses a significant challenge for cross-sensory studies (Laurienti et al. 2002; Raij et al. 2010).

Therefore, to better understand the mechanisms of sensory integration during tactile stimulation, we conducted quiet fMRI experiments in awake and anesthetized rats to investigate the activity within and beyond the whisker-mediated tactile system. We hypothesized that if the neuroimaging approach was sensitive enough, a large amount of data should reveal novel insights into cross-sensory interactions. Specifically, we aimed to (i) characterize the non-core brain-wide regions responding to unisensory whisker pad stimulation, (ii) study how the core and non-core regions respond to varying whisker pathway inputs, and (iii) evaluate whether light anesthesia confounds the detection of cross-sensory activity. To achieve this, we utilized a recently developed zero echo time (zero-TE) fMRI approach, Multi-Band SWeep Imaging with Fourier Transformation (MB-SWIFT) (Lehto et al. 2017), which is a quiet and body movement-tolerant pulse sequence (Paasonen et al. 2020, 2022), making it ideal for fMRI studies in awake animals. This zero-TE approach provides distortion-free (Lehto et al. 2017; Paasonen et al. 2020) high-quality whole-brain coverage, including regions near tissue-air interfaces, brain stem, and cerebellum (Paasonen et al. 2022; Dvořáková et al. 2024). Importantly, zero-TE fMRI provides a robust proxy for neuronal activity (Valjakka et al. 2025).

For clarity, key terms used in this report are defined as follows: “core” refers to the primary sensory system, ie the whisker-mediated tactile system; “non-core” refers to regions outside the whisker-mediated tactile system; “cross-sensory” refers to activity or interactions in non-core systems evoked by the input to the core system; “high-order” refers to cortical associative areas; “low-order” refers to the sensory circuits up to the primary cortices.

## Materials and methods

### Animals

Animal procedures were approved by the National Animal Experiment Board (license ESAVI-2019-028408) and conducted in accordance with the European Commission Directive 2010/63/EU guidelines. 13 adult (7 males and 6 females; 250–430 g at the onset of the first fMRI scan) Sprague–Dawley rats (Envigo RMS B.V., Horst, Netherlands) were used in 54 fMRI sessions in either awake state or under light isoflurane-medetomidine (Iso + Med) anesthesia to obtain data during 1,600 stimuli blocks. No data were excluded from the analyses. On average, 1 rat underwent 4 fMRI sessions, 2 of which were in awake state and 2 under anesthesia. Details on weight, age, and number of experiments under different conditions are provided in Supplementary Table S1.

Rats were group-housed prior to procedures and individually housed during the experiments to minimize damage to the head-implant and surgical wound. Rats were maintained on a 12/12 h light–dark cycle at 22 ± 2°C with 50% to 60% humidity. Food and water were available ad libitum. All experiments were carried out between 8 a.m. and 5 p.m.

### Head-fixation implant surgery

To obtain high-quality fMRI data without significant movement artifacts, a chronic head-fixation implant was attached to the top of the skull of the rats. The implant was shaped to be compatible with a single-loop 22-mm inner diameter MRI coil. Surgical procedures followed previously described methods (Paasonen et al. 2020, 2022). Briefly, rats were anesthetized with isoflurane (Attane vet 1000 mg/g, Piramal Healthcare UK Limited, Northumberland, UK; 5% induction and 2% maintenance) in 30/70% O_2_/N_2_ carrier gas. The scalp was removed from the top of the skull, and anchoring screws were inserted above the olfactory bulb and cerebellum. Four tungsten wire electrodes were placed bilaterally over S1bf for electrophysiological recordings, but data from these were not included in the current study. Layers of bone (Palacos R + G, Heraeus Medical, Hanau, Germany) and dental (Selectraplus, DeguDent GmbH, Hanau, Germany) cements were applied, leaving 2 pins (2-mm diameter) covered with heat-shrinkable tubes passing through the dental cement. After the hardening of the cement, the pins were removed, leaving 2 holes for head-fixation. Buprenorphine (0.03 mg/kg s.c.; Temgesic, Indivior Europe Ltd, Dublin, Ireland), carprofen (5 mg/kg s.c.; Rimadyl, Zoetis Belgium SA, Louvain-la-Neuve, Belgium) and saline (10 mL/kg/day s.c.) were given twice a day for 2 days for analgesia and rehydration. After the surgery, rats were allowed to recover for 1 to 3 weeks before starting habituation protocol.

### Habituation protocol for awake measurements

The 2-week habituation protocol followed our previous protocols (Stenroos et al. 2018; Paasonen et al. 2020, 2022). Briefly, 1 to 3 rats per day were gradually habituated to body restraint and head-fixation. In the first week, rats were allowed to familiarize themselves with the handling person, the sounds of the fMRI sequence, short (≤ 60 s) head and body immobilization times, and positive rewards (almond flakes or 1% sucrose water). On day 5 of the first week, rats were gently immobilized with an elastic plastic foam and a custom-made head-fixation apparatus and kept in the imaging holder for 10 min (Stenroos et al. 2018). Light isoflurane maintenance anesthesia (1.3% to 1.8%) was used while inserting ear plugs and while positioning or removing the rat from the imaging holder. After a weekend break, habituation was continued with progressively longer sessions in the imaging holder, from 10 to 25 min. Rats were also familiarized with masking tape on the whiskers and air puffs used to induce the mechanical whisker movement.

### Isoflurane + medetomidine anesthesia protocol

In anesthesia experiments, rats were lightly anesthetized with a combination of isoflurane (Piramal Healthcare UK Limited, Northumberland, UK) and medetomidine (Domitor vet 1 mg/ml, Orion Corporation, Espoo, Finland). At the beginning of each session, anesthesia was induced with 5% and maintained with 2% isoflurane. A stainless-steel cannula was placed subcutaneously in the back of the animal for the delivery of medetomidine by an infusion pump (AL-1000, World Precision Instruments, Friedberg, Germany). A medetomidine bolus (0.015 mg/kg, s.c.) was given 12.9 ± 4.4 min (*n* = 26) after the induction of isoflurane anesthesia, followed by infusion (0.03 mg/kg/h s.c.) 15 min later. After the medetomidine bolus, isoflurane was gradually reduced (typically to 0.3% to 0.5%) to achieve a respiration rate of 45–65 breaths per minute. The first fMRI scan started 48 ± 14 min (*n* = 26) after the medetomidine bolus, giving time for the level of anesthesia to stabilize. After measurements, atipamezole (0.5 mg/kg s.c.; Antisedan vet 5 mg/ml, Orion Corporation, Espoo, Finland) was administered, and rats were returned to their cages.

### Whisker pad stimulation protocols

Throughout this report, “whisker pad stimulation” refers to either mechanical or electrical stimulation aiming to activate the whisker-mediated tactile system. Stimuli were given with a stimulus generator (STG4008-16 mA, Multi-Channel Systems MCS GmbH, Reutlingen, Germany; MC_Stimulus II software V 3.5.11), applied unilaterally to the left or right with the general aim to produce equal amounts of data for both sides.

A non-invasive mechanical air-puff mediated whisker deflection (20° to 40°) was used in both anesthetized and awake conditions. To control the potential effects of the acoustic sound induced by the mechanical stimulation setup but to prevent discomfort from needles, electrical stimulation was used only under anesthesia. For electrical stimulation, stainless steel 30G needle electrodes were placed between whisker row pairs A–B and C–D. Each electrical stimulation pulse consisted of 2 mA for 300 μs followed by −2 mA for 300 μs.

In the air puff-based mechanical stimulation, posterior-to-anterior deflections were achieved with 5-ms air puffs directed at adhesive tape (20 × 15 mm) attached to a maximal tuft of the whiskers. Pressurized air (1.0 to 1.5 bar) was gated by a solenoid valve (custom-made by Neos Biotec, Pamplona, Spain), controlled by the stimulus generator. The air from the solenoid valve was directed to the posterior side of the whiskers via a 7-m pneumatic pipe (6-mm inner diameter) and a 50-cm flexible tube (3-mm inner diameter). To achieve a reliable deflection of the whiskers (typically 20° to 40°), the angle of the tube end was fine-tuned for each experiment separately. Slow-motion video recordings confirmed that the approach reliably deflected whiskers up to 20-Hz frequency.

All stimulation protocols consisted of 16-s stimuli blocks interleaved with 44-s breaks. The stimuli analyzed here were given at either 1, 5, 9, 13, or 17 Hz frequency. Additionally, 3 Hz and 7 Hz stimuli were given under anesthesia but were not included in the current study since these frequencies were left out from awake experiments to minimize the scanning time. Each frequency was repeated 4 times, leading to 20 1-min stimulus and baseline blocks per scan for the current analysis. The order of the different frequencies and their repetitions within each experiment was randomized, as was also the order of mechanical and electrical protocols in the experiments conducted under anesthesia.

Natural whisking (1 to 25 Hz) can be roughly divided into slower large-amplitude (5 to 10 Hz) (Sofroniew and Svoboda 2015; Adibi 2019) and faster small-amplitude (15 to 25 Hz) (Adibi 2019) whisking. Preliminary analysis (Supplementary Fig. S1) indicated similar fMRI responses for 5 and 9 Hz, and for 13 and 17 Hz stimuli, aligning with the natural whisking categories. Therefore, to simplify the representation of the results, stimuli were grouped into low- (1 Hz), mid- (5 or 9 Hz), and high-frequency (13 and 17 Hz) categories. However, most analyses were also performed using individual stimulation frequencies, with results provided in the supplementary material.

### Magnetic resonance imaging

The MRI data acquisition was similar to that described earlier (Paasonen et al. 2022). Briefly, data were acquired in a 9.4 T 31-cm bore magnet with 12-cm inner diameter gradient coil set, interfaced with an Agilent DirectDRIVE console (Palo Alto, CA, USA). A linear 22-mm inner diameter surface coil (Neos Biotec, Pamplona, Spain) was used for both transmission and receiving the radio-frequency signals.

Anatomical reference images were acquired with an MB-SWIFT sequence with the following parameters: 4,000 spokes per spiral, 16 stacks of spirals, repetition time 3 ms for a spoke, 4 radiofrequency pulses per spoke, flip angle 5° to 6°, excitation/acquisition bandwidths of 192/384 kHz, matrix size 256^3^, field of view 40^3^ mm, and 156 μm isotropic resolution. To increase anatomical contrast, a magnetization transfer pulse (γB1 = 125 Hz, offset 2,000 Hz, duration of 20 ms) was given at every 32 spokes. The sequence had a total acquisition time of ~ 4 min.

Functional data were obtained with an MB-SWIFT sequence with the following parameters: 2047 spokes per spiral, 1 spiral, repetition time 0.97 ms for a spoke, 4 radiofrequency pulses per spoke, flip angle 5–6°, excitation/acquisition bandwidths of 192/384 kHz, matrix size 64^3^, field of view 40^3^ mm, and 625 μm isotropic resolution. The field-of-view covered the whole brain, including the cerebellum and olfactory bulb. The acquisition time for a single fMRI volume was 2 s. The first stimulus block was preceded by a 3-min baseline period to ensure the stabilization of baseline. Subsequently, the lengths of awake and anesthesia fMRI scans were 690 (23 min) and 930 volumes (31 min), respectively. The first 2 volumes were discarded as the signal was reaching a steady-state.

A physiology monitoring equipment (Model 1025, Small Animal Instruments Inc., New York, NY, USA) was used to follow the breathing rate. In anesthetized animals, the breathing rates were 48 ± 9 per minute (*n* = 52) at the onset of the fMRI scans. In awake animals, the breathing rates were 108 ± 14 per minute (*n* = 28) at the onset of the scan, and varied up to 144 ± 22 per minute during the 23-min fMRI measurement. The temperature of anesthetized rats was monitored with a rectal probe. A warm water circulation system (Corio CD, Julabo, Seelbach, Germany) was used to keep the animals warm (36.9 ± 0.6°C, *n* = 51, measured at the onset of the anesthesia fMRI scans). A video camera (12 M-i, MRC Systems GmbH, Heidelberg, Germany) was used to monitor the whisker deflections and animal behavior during the MRI.

### Data processing and analysis

Data processing and analysis shared similarities with our previous work (Paasonen et al. 2020, 2022). A Snakemake (https://snakemake.readthedocs.io) script was used for (i) reconstruction of images into NIfTI, (ii) saving data in the BIDS structure (https://bids.neuroimaging.io), (iii) co-registration, and (iv) motion correction. The MB-SWIFT (Idiyatullin et al. 2015) data were reconstructed using an in-house Python (version 3.10) script for a center-out data re-gridding and iterative FISTA algorithm (Beck and Teboulle 2009), performed volume-by-volume with 13 iterations. Representative unprocessed fMRI images are shown in Supplementary Fig. S2. The reconstructed anatomical brain volumes were co-registered to a study-specific template using N4 bias correction, and linear and non-linear SyN registrations of Advanced normalization tools (ANTs, http://stnava.github.io/ANTs).

Even though the rats were head-fixed, which diminished movement of the skull (Supplementary Video S1), the fMRI data were motion-corrected with a tailor-made Python script using masking, N4 bias correction, and ANTs rigid co-registration of each volume to the first volume of the 4D data set. Motion correction parameters, indicating no major movement-induced confounding effects (Supplementary Figs. S3 and S4), were saved for further use. The motion-corrected fMRI images were mirrored in the left–right orientation, if needed, to locate the stimulation in the same side in all data sets. Subsequently, the fMRI images were translated to the template reference frame for group-level analyses using linear and non-linear transformations from the anatomical co-registration. No spatial smoothing or global signal regression was applied to the fMRI data.

The preprocessed zero-TE fMRI data were analyzed using conventional methods. First-level statistical maps (β-values) for each dataset were computed using a standard general linear model-based FLAME in the FSL FEAT toolbox, testing whether stimulus responses significantly differed from the baseline. A mask covering the whole brain was used. Stimulus duration was set to 16 s for the main results and 4 s for selected supplementary analyses. The parameters used for the hemodynamic response function with the general linear model were derived in separate experiments (Valjakka et al. 2025). At the individual experiment level, each stimulation frequency was treated as an event, resulting in 4 stimulus blocks for each event. Motion parameters from the rigid motion correction were used as confounding regressors. Group-level voxel-wise maps were produced by concatenating experiment-level maps and testing whether voxel intensities in the stimulus response maps significantly differed from zero (1-sample *t*-test). The statistical tests were performed with the Randomize (Winkler et al. 2014) tool while taking multiple comparisons into account by using the family-wise error (FWE) correction. FreeSurfer (Freeview, version 2.0, https://surfer.nmr.mgh.harvard.edu/) and FSLeyes (FSL version 6.0.5, https://fsl.fmrib.ox.ac.uk/fsl/fslwiki) were used for the evaluation and representation of the statistical maps. To maintain a narrow scope, the current work focuses on positive responses, whereas negative responses are discussed only briefly.

Regions of interest (ROIs) were drawn with FSLeyes on the fMRI reference frame according to an anatomical atlas (Paxinos and Watson 2014), aiming to minimize contribution from ventricles and large blood vessels. Regions were taken for further analyses if they included significant (*P* < 0.005, FWE-corrected) voxels with any stimulation frequency range or experimental group. To minimize the potential effect of motion, the fMRI time series were regressed using motion correction parameters (see Supplementary Fig. S5 for awake data). Subsequently, ROI time series were computed as an intensity average under the ROI, temporally smoothed, and grouped according to different frequency ranges and experimental groups.

The average response values for each ROI were calculated from an 11-volume (22-s) window starting at the onset of the 16-s stimulus. An exception of a shorter 5-volume (10-s) window was used for SC due to its complex biphasic response shape, where the 11-volume window resulted in close-to-zero values, not reflecting the underlying response. The stimulation frequency-dependence of fMRI responses was investigated by fitting a linear function on the average responses of different stimulation frequency ranges, assuming the pools to be equidistant from each other. The slope from linear fitting was used in determining whether the fMRI response displayed an association with stimulation frequency. The linearity of association was determined by testing the normality of the residuals. Lastly, the categorization of different fMRI response profiles and temporal signal characteristics was performed using hierarchical clustering with the linkage-function from the Python scipy-module. Briefly, correlation matrices were first computed for individual responses to assess their similarity (or correlation). Subsequently, the response profiles or time series were grouped into clusters using an agglomerative approach: each profile or time series began as its own cluster, and at each step, the 2 most similar clusters (ie those with the highest correlation) were merged. This process was repeated until a stopping criterion was met (for example, when only 4 clusters remained). Clusters consisting of a single region and high hierarchical level were excluded from further analysis and discussion.

Group-level values are represented as group-level mean ± standard deviation unless stated otherwise. The types of statistical tests used are mentioned in the Results section and in the figure legends. The threshold for statistical significance was set to *P* < 0.005 in voxel-wise statistical maps, and elsewhere to *P* < 0.05, unless otherwise stated. Corrections for multiple comparisons were performed with FWE-correction, unless stated otherwise.

## Results

### Awake rats exhibit the largest whisker pad stimulation-induced signal changes outside the whisker-mediated tactile system

**Fig. 1:**
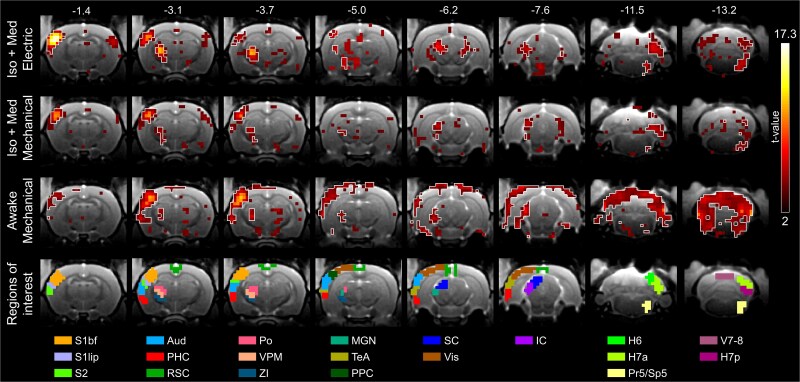
Group-level statistical maps showing significant responses to mid-frequency whisker pad stimuli across 8 representative slices. White outlines on the statistical maps indicate the regions with *P* < 0.005 (FWE-corrected), while the color maps are thresholded with *t*-value > 2. The results are obtained from 208–224 stimulus blocks during 26–28 scans in each group. Values in the top row indicate the approximate distance from bregma for each slice. Statistical maps are overlaid on high-resolution anatomical images. A whole-brain view with 22 slices is shown in Supplementary Fig. S6. H6, hemisphere of cerebellar lobule 6 (simplex); H7a, anterior hemisphere of cerebellar lobule 7 (crus 1 and 2); H7p, posterior hemisphere of cerebellar lobule 7 (paramedian 1); Iso + med, isoflurane and medetomidine anesthesia; Po, posterior thalamic nuclei; Pr5/Sp5, principal trigeminal nuclei and spinal trigeminal nuclei; RSC, retrosplenial cortex; S1bf, primary somatosensory cortex, barrel field; S1lip, primary somatosensory cortex, lip region; TeA, temporal association cortex; V7–8, vermis of cerebellar lobules 7 and 8; VPM, ventral posteromedial thalamic nuclei.

**Fig. 2:**
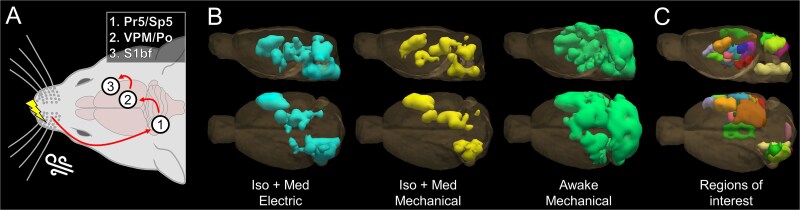
Schematic illustration of the whisker-to-barrel cortex core pathway activated by either mechanical (air puff) or electrical stimulus (a), a 3D illustration of combined significant voxels (*P* < 0.005, FWE-corrected) from low-, mid-, and high-frequency analyses within each group (b), and a 3D presentation of ROIs used in the subsequent analyses (c). The results are obtained from 1,600 16-s stimulation blocks given during 54 sessions (520–560 stimuli blocks per group). The colors for ROIs in C correspond to those in Fig. 1.

Group-level statistical maps to mid-frequency whisker pad stimuli are shown in Fig. 1 and Supplementary Fig. S6. Regardless of the stimulus approach or wakefulness, signal changes were observed in key nodes of the whisker-mediated tactile system, including ipsilateral principal and spinal trigeminal nuclei (Pr5/Sp5), contralateral ventral posteromedial (VPM) and posterior (Po) thalamic nuclei, and contralateral S1bf. A schematic illustration of these key nodes is shown in Fig. 2a. Signal changes were also detected in other parts of the whisker-mediated tactile system (Adibi 2019), such as in S2, as well as in the lip region of primary somatosensory cortex (S1lip), likely indicating non-whisker-related tactile input from the snout.

Signal changes (Fig. 1) were also detected in auditory areas, including auditory cortex (Aud), MGN, and IC, and in visual areas such SC. ZI, involved in sensory integration and sensory-motor processes (Wang et al. 2020), and multiple cerebellar regions, including the hemisphere of lobule 6 ie simplex (H6), the anterior hemisphere of lobule 7 ie crus 1–2 (H7a), and the posterior hemisphere of lobule 7 ie paramedian 1 (H7p), also displayed significant signal changes in all groups. In awake rats, cortical signal changes extended to PPC, retrosplenial (RSC), visual (Vis), temporal association (TeA), and parahippocampal (PHC) cortices, as well as to the cerebellar vermis of lobules 7 and 8 (V7–8), which contain a representation of the face (Manni and Petrosini 2004) and may therefore exhibit partially stimulus-unspecific activity. The signal changes in the cerebellum appeared bilaterally in awake rats. Notably, PPC exhibited signal changes in anesthetized rats only after electrical stimulation. In summary, awake rats exhibited widespread signal changes across somatosensory, auditory, visual, high-order, and cerebellar regions, while anesthetized rats showed more restricted responses, particularly in the high-order cortical and cerebellar regions.

Figure 2B and Supplementary Video S2 provide 3D illustrations of significant signal changes (*P* < 0.005, FWE-corrected) across low-, mid-, and high-frequency stimulation analyses. Altogether, signal changes covered 2,234 voxels in awake rats, compared to 729 voxels with electrical stimulation and 469 voxels with mechanical stimulation under anesthesia, emphasizing the restricted spatial coverage of signal changes in anesthetized conditions. Supplementary Fig. S7 presents analogous 3D statistical maps for each group and stimulation frequency range. Supplementary Fig. S8 shows statistical maps generated using only subsets of the data, demonstrating that a large number of stimuli (> 200) was necessary to localize many of the effects outside S1bf. Supplementary Fig. S9 illustrates the extent of statistical maps at the subject level, including a representative map from a single subject in each condition.

To investigate further the frequency-dependency and temporal characteristics of fMRI responses, ROIs (Fig. 1, Fig. 2c, Supplementary Video S2) were defined based on the localization of the signal changes (Fig. 2b) in an anatomical atlas (Paxinos and Watson 2014) and their potential structural or functional connections to the whisker-mediated tactile system. Table 1 lists the selected ROIs and their abbreviations, showing significant voxels in all ROIs in awake animals, but only in 15/20 and 14/20 ROIs with electrical and mechanical stimulation under anesthesia, respectively.

**Table 1:** Regions of interest derived from the statistical maps and literature search.

		Region of interest	Iso + med electrical	Iso + med mechanical	Awake mechanical	Abbreviation
**Core regions**	Whisker-mediated tactile system	Principal trigeminal nuclei and spinal trigeminal nuclei	**+**	**+**	**+**	Pr5/Sp5
		Ventral posteromedial thalamic nuclei	**+**	**+**	**+**	VPM
		Posterior thalamic nuclei	**+**	**+**	**+**	Po
		Primary somatosensory cortex, barrel field	**+**	**+**	**+**	S1bf
		Secondary somatosensory cortex	**+**	**+**	**+**	S2
**Non-core regions**	High-order regions	Parahippocampal cortex	**−**	**−**	**+**	PHC
		Posterior parietal cortex	**+**	**−**	**+**	PPC
		Retrosplenial cortex	**−**	**−**	**+**	RSC
		Temporal association cortex	**−**	**−**	**+**	TeA
	Auditory regions	Auditory cortex	**+**	**+**	**+**	Aud
		Inferior colliculus	**+**	**+**	**+**	IC
		Medial geniculate nuclei	**+**	**+**	**+**	MGN
	Visual regions	Superior colliculus	**+**	**+**	**+**	SC
		Visual cortex	**−**	**−**	**+**	Vis
	Cerebellum	Hemisphere of lobule 6 (simplex)	**+**	**+**	**+**	H6
		Anterior hemisphere of lobule 7 (crus 1 and 2)	**+**	**+**	**+**	H7a
		Posterior hemisphere of lobule 7 (paramedian 1)	**+**	**+**	**+**	H7p
		Vermis of lobules 7 and 8	**−**	**−**	**+**	V7–8
	Other	Primary somatosensory cortex, lip region	**+**	**+**	**+**	S1lip
		Zona incerta	**+**	**+**	**+**	ZI

Awake animals exhibited signal changes (*P* < 0.005, FWE-corrected) in all Regions of interest (ROIs) (indicated by +), while only 15/20 and 14/20 ROIs contained significant voxels in response to electrical or mechanical stimulation in anesthetized rats, respectively. In the subsequent evaluation, the ROIs were grouped into either core or non-core regions, and further divided into whisker-mediated tactile system, high-order regions, auditory regions, visual regions, and cerebellum.

Despite several high-order regions showed activity in awake animals, no significant voxels were observed in the frontal brain (Fig. 1 and 2), which is known to support various cognitive functions. However, when a shorter 4-s stimulus duration was used in the GLM analyses, signal changes (*P* < 0.05, FWE-corrected) became visible around the anterior cingulate area in awake but not in anesthetized animals (Supplementary Fig. S10). These observations indicate that the temporal response profiles to stimuli may vary across brain regions, making it challenging to capture all relevant activity using the simple, conventional analysis applied here.

Negative responses to whisker pad stimuli are shown in Supplementary Fig. S11. No significant voxels were observed with mechanical stimulation in the anesthetized rats. In contrast, electrical stimulation elicited negative signal changes in the striatum, while awake animals exhibited negative responses in frontal cortical, thalamic, and hypothalamic regions.

### The fMRI responses in the whisker-mediated tactile system increase with stimulation frequency in awake but not in anesthetized rats

**Fig. 3:**
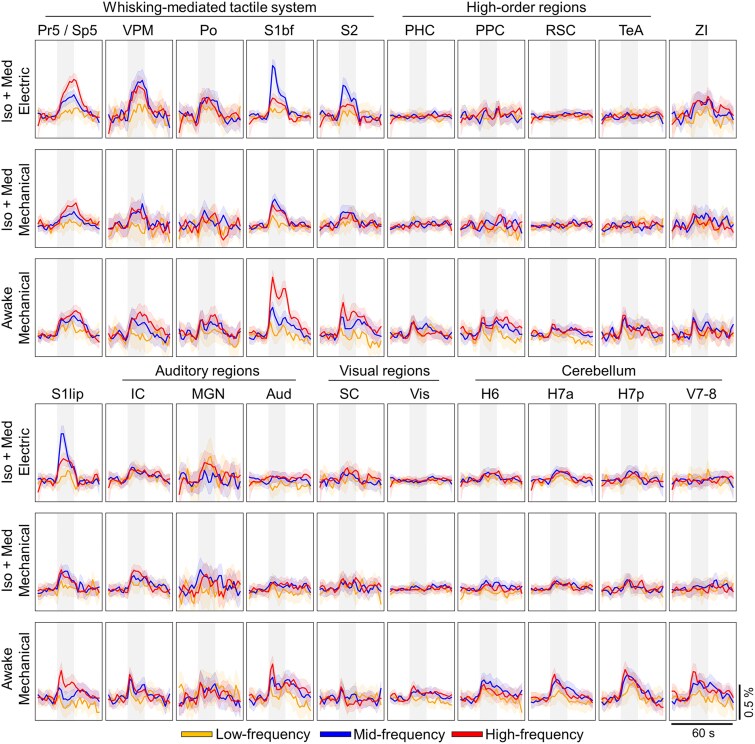
Group-level mean time series for each ROI. Each average time series is a result of 104–224 stimuli (see Supplementary Table S1)**.** The shaded vertical gray region indicates the timing for the 16-s stimulus block. The list of abbreviations for ROIs can be found in Fig. 1 and in Table 1. The 90% confidence interval is shown as a shaded region around the mean time series.

**Fig. 4:**
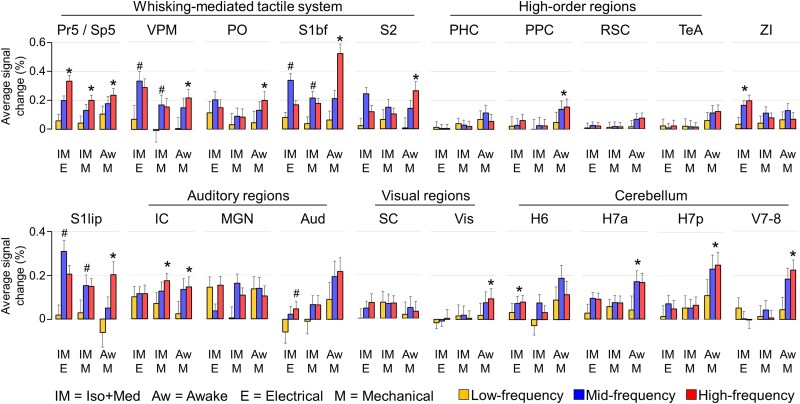
Group-level average fMRI response in each group and ROI. The average response was typically calculated over a 20 s window starting from the stimulus onset (see materials and methods). For each ROI and group, we tested (i) whether the slope determined by stimulation frequency and average response deviated from 0 (*t*-test), and (ii) whether the relationship between the stimulation frequency and average response was linear (normality test for residuals of the fit). Asterisk (*) denotes a significant linear slope (*t*-test for slope *P* < 0.05, normality test *P* > 0.05), and hash (^#^) denotes a significant non-linear slope (*t*-test for slope *P* < 0.05, normality test *P* < 0.05). The full list for uncorrected p-values can be found in Supplementary Table S2. The error bars indicate the 90% confidence interval. The list of abbreviations for ROIs can be found in Fig. 1 and in Table 1.

To study the positive fMRI signal characteristics during the whisker pad stimuli, group-level average time series (Fig. 3; see Supplementary Fig. S12 for all stimulation frequencies and Supplementary Fig. S13 for functional signal-to-noise ratios) were extracted from the selected ROIs (Table 1). As a measure of caution, we verified that the average time series remained similar even when smaller, randomly selected sample sizes were used (Supplementary Fig. S14). Average fMRI responses were derived from time series, and significance and linearity for the slopes between the stimulation frequency and response strength were estimated (Fig. 4). Supplementary Fig. S15 visualizes the linearity of responses across all stimulation frequencies.

A significant linearly increasing response (*t*-test for slope *P* < 0.05, normality test *P* > 0.05) to low-, mid-, and high-frequency stimuli was detected in Pr5/Sp5 in all groups (Figs. 3 and 4, Supplementary Table S2). This finding confirms that the input into the first relay station of the whisker-mediated tactile system was linearly modulated with the applied stimulation protocol. Minimal adaptation is expected in trigeminal ganglion neurons (Adibi and Lampl 2021) and Sp5 (Devonshire et al. 2012) with stimulation frequencies up to 18 to 20 Hz (17 Hz highest in the current work).

Although fMRI responses in brain stem showed linear increases, this was not observed in thalamus in all groups (Fig. 4, Supplementary Table S2). In awake rats, both VPM and Po exhibited linear responses (*t*-test for slope *P* < 0.05, normality test *P* > 0.05) to the whisker pad stimulation. However, in anesthetized rats, VPM showed a non-linear relationship (*t*-test for slope *P* < 0.05, normality test *P* < 0.05) in anesthetized rats (Fig. 4), while Po displayed no significant slope, highlighting the confounding effects of anesthesia, as also suggested earlier (Petersen 2007). A similar but more pronounced pattern was seen in cortical areas S1bf and S2, where the slope between stimulation frequency and fMRI response was linear in awake rats, but either non-linear or non-significant in anesthetized rats.

Taken together, the fMRI responses in all key nodes of whisker-mediated tactile system followed the stimulation frequency in awake rats. In anesthetized rats, both the thalamic and cortical regions showed saturated fMRI responses, peaking already with the mid-frequency stimulation. These observations indicate the confounding effect of Iso + Med anesthesia on the signaling in the whisker-mediated tactile system.

### The fMRI responses in non-core regions are weak in amplitude

As described above, several regions outside the whisker-mediated tactile system showed reliable signal changes in response to the whisker pad stimuli (Fig. 1). However, as shown in Figs. 3 and 4, the signal changes in non-core regions were low in amplitude. Under anesthesia, the key nodes of whisker-mediated tactile system (Pr5/Sp5, VPM, and S1bf) exhibited significantly higher average response strengths in comparison to auditory (*P* < 0.001 with electrical and *P* = 0.039 with mechanical stimulation, 2-sample Student’s *t*-test), visual (*P* < 0.001 with both electrical and mechanical stimulation, 2-sample Student’s *t*-test), and cerebellar (*P* < 0.001 with both electrical and mechanical stimulation, 2-sample Student’s *t*-test) regions. In awake rats, the average signal changes were significantly smaller in the auditory (*P* = 0.016, 2-sample Student’s *t*-test), visual (*P* < 0.001, 2-sample Student’s *t*-test), and high-order regions (*P* < 0.001, 2-sample Student’s *t*-test) but not in cerebellar regions (*P* = 0.081, 2-sample Student’s *t*-test) as compared to the key nodes of the core pathway. These observations confirm one of our initial hypotheses that the signal changes in many non-core regions in response to whisker pad stimuli are weak but nonetheless can be reliably detected with larger data sets.

### Responses in most non-core regions do not increase with higher stimulation frequencies

Another notable observation in Figs. 3 and 4 is that the majority of the auditory, visual, and high-order regions did not exhibit a significant slope between stimulation frequency and fMRI response strength. Among the high-order regions in awake rats, only PPC showed linear slope between the two variables, while in RSC, PHC, and TeA no significant slope was observed (Fig. 4). In SC (visual system) and MGN (auditory system), no significant slope was observed in any of the groups. Similarly, Aud showed no significant slope with mechanically stimulated awake and anesthetized rats. However, IC exhibited a significant linear slope with mechanical but not with electrical stimulation. This may suggest that the response in IC was modulated by the audible noise from the mechanical stimulation setup. Nevertheless, the results obtained with silent electrical stimulation indicate that the stimulus-induced responses in IC did not correlate with the stimulation frequency, similar to the observations in SC observed in all groups. In summary, the responses in most of the auditory, visual, and high-order regions did not follow the responses in the whisker-mediated tactile system, regardless of the state of wakefulness or stimulus type.

In contrast, most of the cerebellar regions in awake rats showed a significant linear slope between the stimulation frequency and the strength of the fMRI response (Fig. 4). This differs from the results obtained in anesthetized animals, where the majority of the cerebellar regions displayed no relationship between input and response. These observations indicate that the interactions between cerebellum and whisker-mediated tactile system had been disturbed by the Iso + Med anesthesia.

### The fMRI response profiles in awake rats within the whisker-mediated tactile system are coherent but differ from non-core regions

**Fig. 5:**
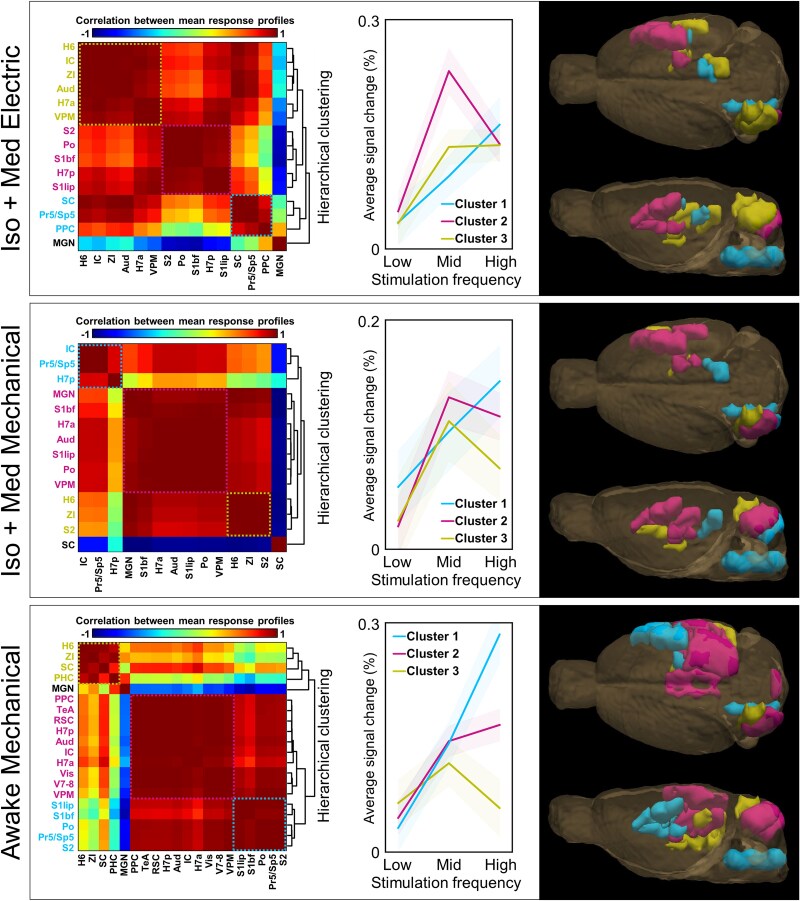
Hierarchical clusters of average fMRI response profiles. The average fMRI response profiles were derived from data shown in Fig. 4, and clustered by using hierarchical clustering. The correlation matrices on the left indicate the similarity between the frequency response profile curves across ROIs. The obtained hierarchical tree (or dendrogram) is shown on the right side of the matrices. Subsequently, average response curves were calculated for 3 main clusters for each group, which are shown in the middle. Clusters with single regions and high hierarchy were excluded from the illustrations, and regions without significant signal changes (Table 1) were left out from the analysis. The localization of the clusters is illustrated on the right. The clustered brain regions are color-coded in matrices, in the response profile graphs, and in the 3D illustrations. The cluster including brain stem nuclei is color-coded with the same color in each group. The shaded region around the average response profiles indicates the 90% confidence interval. The list of abbreviations for ROIs can be found in Fig. 1 and in Table 1.

We next examined more closely the similarities in the response profiles to different stimulation frequencies across the ROIs. For this purpose, a 3-point response profile, namely the fMRI average response plotted against the stimulation frequency, was derived from the data shown in Fig. 4. Subsequently, the response profiles were hierarchically clustered, as shown in Fig. 5 (see Supplementary Fig. S16 for clustering results obtained with all stimulation frequencies).

Consistent with previous findings, clusters including Pr5/Sp5 (clusters #1, Fig. 5) showed a linear increase in the response strength to the stimulation frequency in each group. In awake animals, this cluster included most of the thalamic and cortical nodes of the whisker-mediated tactile system, which were absent in corresponding clusters in anesthetized animals. Despite spatial proximity, non-core regions in awake rats did not cluster with the primary pathway but instead formed separate clusters with distinct frequency-modulation profiles. In anesthetized rats, particularly after mechanical stimulation, thalamic and cortical nodes of the whisker-mediated tactile system often clustered with non-core regions (clusters #2, Fig. 5). The results obtained in awake rats strengthen the conclusion that the stimulation frequency-dependent modulation clearly differs between core and non-core regions, possibly reflecting the fundamental differences between primary sensory and cross-sensory processing. However, it was difficult to draw the same conclusion in anesthetized animals. The main findings remained consistent even when only half of the data were used (Supplementary Fig. S17).

### The temporal dynamics of non-core regions differ from those in the whisker-mediated tactile system in awake rats

**Fig. 6:**
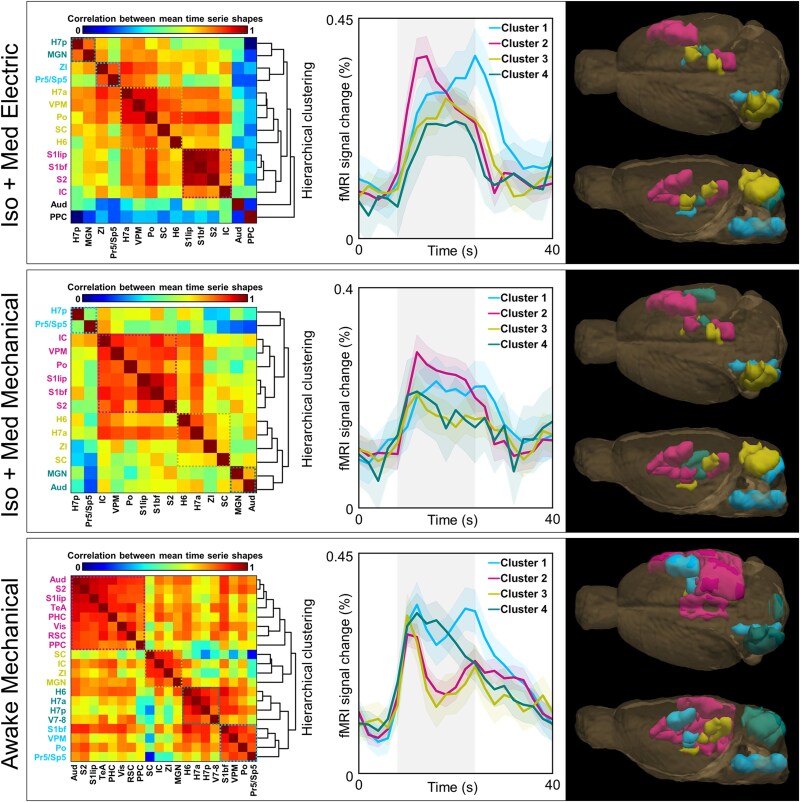
Hierarchical clusters of average fMRI time series. When studying the temporal characteristics of the signal changes, fMRI time series were averaged across all stimulation frequencies and clustered by using hierarchical clustering. The correlation matrices on the left indicate the similarity between time series across ROIs. The obtained hierarchical tree (or dendrogram) is shown on the right side of the matrices. Subsequently, average time series were calculated for 3 to 4 main clusters for each group, which are shown in the middle. Clusters with single regions and high hierarchy were excluded from the illustrations, and regions without significant signal changes (Table 1) were omitted from the analysis. The localization of the clusters is illustrated on the right. The clustered brain regions are color-coded in matrices, in the response profile graphs, and in the 3D illustrations. The cluster including brain stem nuclei is color-coded with the same color in each group. The shaded vertical gray region indicates the timing of the 16-s stimulus block. The shaded region around the average time series indicates the 90% confidence interval. The list of abbreviations for ROIs can be found in Fig. 1 and in Table 1.

We further extended our analyses to study the temporal characteristics of the fMRI signals by hierarchically clustering the ROI-specific average time series (Fig. 6). Overall, the categorization of ROIs into clusters based on their temporal profiles was vague in anesthetized animals. With electrical stimulation, cluster #2 represented mostly cortical, and clusters #3 and #4 thalamic and cerebellar temporal signals, but no clear anatomical or functional clusters appeared with mechanical stimulation. In awake rats, however, distinct clusters were observed for the primary pathway (cluster #1, Fig. 6), cortical auditory, cortical visual, and high-order regions (cluster #2, Fig. 6), subcortical auditory and visual regions (cluster #3, Fig. 6), and cerebellum (cluster #4, Fig. 6). Interestingly, auditory, visual, and high-order regions exhibited a similar initial rise to core regions, but a fast decay to a lower amplitude level after only 4 to 6 s of stimulation, whereas a sustained high-amplitude response was maintained in the key nodes of whisker-mediated tactile system during the stimulus. Moreover, the cerebellar regions displayed a distinct profile compared to the regions in cerebrum and brain stem. These observations indicate that also the temporal dynamics of fMRI signal differ between the core and non-core regions during the sensory processing in awake rats. Importantly, these features were not visible under Iso + Med anesthesia. The main findings were reproducible even with a smaller sample size (Supplementary Fig. S18).

There were a few additional observations worthy of consideration. First, the signal rise to peak value appeared slower under anesthesia (4 to 8 s) in comparison to that in awake rats (2 to 4 s), consistent with earlier reports of a slower hemodynamic response under anesthesia (Gao et al. 2017; C. Martin et al. 2006a). Second, the awake rats exhibited a biphasic hemodynamic signal to whisker pad stimulation, characterized by an initial peak and followed by a delayed secondary peak (except in the cerebellum). This delayed peak was not observed in anesthetized rats, although biphasic responses have been reported under anesthesia in different experimental conditions (Gil et al. 2024; Grimm et al. 2024). Nevertheless, similar hemodynamic responses to whisker pad stimulation in both awake and anesthetized rats have been reported earlier (Martin et al. 2013b). The delayed peak likely originates from the slower components of functional hyperemia during prolonged stimulation (Institoris et al. 2022; Ahn et al. 2023), which may be disturbed by anesthesia (Thrane et al. 2012).

## Discussion

To our knowledge, this is the first study to detect brain-wide cross-sensory activity in response to whisker pad stimulation in the rat brain. Additionally, it is the first to identify the stimulation frequency-independence of responses in non-core regions. Importantly, we also demonstrated that even light anesthesia not only affected the evoked activity in the primary sensory pathway but also disrupted the large-scale cross-sensory activity to a unisensory stimulus.

### Brain-wide activity evoked by unisensory stimulus

The most typical finding from whisker pad stimulation fMRI studies is activation in contralateral S1bf (Yangt et al. 1996; Lu et al. 2003, 2005; Sachdev et al. 2003; De Celis Alonso et al. 2008; Sanganahalli et al. 2008, 2022; de Celis Alonso et al. 2012; Devonshire et al. 2012; Yu et al. 2012). While some studies have also detected activity in other key nodes, such as in thalamus (VPM or Po) (Devonshire et al. 2012; Sanganahalli et al. 2022) or brain stem (Sp5) (Devonshire et al. 2012), none have reported signal changes in non-core regions. A few factors could explain the differing outcomes between this and previous studies. First, the echo planar imaging protocols used in previous experiments may have been suboptimal because of the limited number of coronal 2D slices as well as issues with sensitivity to image distortions. These limitations can restrict spatial coverage, possibly excluding many brain regions detected here. Second, previous work may have focused on a single sensory system or region, potentially overlooking signal changes in other relevant brain areas. Third, as observed in the present work, the signal changes in the non-core regions are weak and may thus be difficult to detect with small sample sizes. Fourth, arbitrary statistical thresholds in neuroimaging analyses may lead to unintentional exclusion of interesting findings (Taylor et al. 2023), which can be particularly detrimental when evaluating the weaker cross-sensory signals.

While no rat fMRI studies have reported such widespread signal changes to whisker pad stimulation as described here, a recent experiment in mice (Dinh et al. 2024) shares many similarities with the current work. In addition to the cortical and thalamic key nodes of the whisker-mediated tactile system, Dinh et al. (2024) reported signal changes in the visual system (SC and Vis) and PPC to electrical whisker pad stimulation. Although not specifically reported, the activation maps also appear to cover parts of Aud (Dinh et al. 2024). These observations indicate that similar cross-sensory responses to whisker pad stimulation can be detected with fMRI in visual, auditory, and integrative systems in both mice and rats.

Several mechanisms have been suggested to explain the cross-sensory interplay. Potential structural connections allowing a cross-sensory information flow involve direct projections between the sensory cortices, inputs from the thalamic sensory tracts to multiple primary sensory cortices, inputs from the periphery to cross-sensory (or multisensory) thalamic nuclei, corticothalamo-cortical tracts between different sensory cortices, and feedback projections from the high-order regions to the cross-sensory cortices (Raij et al. 2010; Sieben et al. 2013; Petro et al. 2017; Gu et al. 2020). As the high-order regions, or association areas, are thought to be involved at least in the integration of sensory information (Yau et al. 2015a), the fMRI responses in these regions are expected. RSC integrates multimodal sensory information (Sugar et al. 2011; Xiang et al. 2023) and has structural connections with the sensory cortices (Aud, S2, Vis), thalamus (MGN, Po), ZI, PPC, PHC, and brain stem (Sugar et al. 2011; Mitchell et al. 2018; Aggleton et al. 2021; Xiang et al. 2023). PHC, including the perirhinal, postrhinal and entorhinal cortices, responds to multiple sensory stimuli (Furtak et al. 2007; Aminoff et al. 2013) and has structural connections with many of the sensory and associative areas listed above (Furtak et al. 2007; Sugar et al. 2011). PPC, traditionally viewed as a high-order hub for modality-specific sensory integration (Ghazanfar and Schroeder 2006a), receives direct projections from the primary sensory cortices, including S1bf (Mohan et al. 2018). The exact physiological function of TeA remains unclear, but it receives inputs from more than a hundred regions, including the several primary sensory regions (Tasaka et al. 2020), suggesting an integrative role. In summary, a simple unisensory stimulus was able to elicit fMRI responses in associative networks throughout the rat brain.

While the cerebellum is primarily associated with motor coordination, many cerebellar regions are specialized in sensory processing (Bosman et al. 2010) and receive inputs from multiple sensory modalities (Tsutsumi et al. 2023). Despite this, the cerebellum is rarely examined in rodent fMRI studies, and no prior fMRI reports describe cerebellar responses to whisker pad stimulation in rats. Several regions in the cerebellar cortex, mainly in the lobules 6–7, receive inputs from the whisker pad (Shambes et al. 1978; Manni and Petrosini 2004; Bosman et al. 2010; Ju et al. 2019; Tsutsumi et al. 2023), and electrophysiological activity in response to whisker pad stimulation has been reported in these areas (Bosman et al. 2010; Ju et al. 2019). Therefore, the evoked cerebellar activity in H6, H7a, and H7p in the current study aligns well with the histological and electrophysiological findings. Interestingly, a recent study revealed bilateral projections from the trigeminal sensory nuclei complex to cerebellar cortex (Tsutsumi et al. 2023), potentially explaining the bilateral fMRI responses observed here. Additionally, the cerebellum receives inputs from the cerebrum (Suzuki et al. 2012). V7–8 are known to receive projections from the somatosensory and cingulate cortices (Suzuki et al. 2012), which can explain the fMRI responses observed in these regions. Alternatively, these responses may reflect motor coordination (Manni and Petrosini 2004) triggered by the stimulation, which in our study would manifest as non-specific activity.

The exact origin of negative fMRI responses remains debated, and such responses are rarely reported following whisker pad stimulation (De Celis Alonso et al. 2008; Martin et al. 2013a). While responses in the striatum, which is part of the whisker-mediated tactile system (Adibi 2019), can be considered relevant, the absence of striatal responses in awake animals suggests that these responses were specific to electrical stimulation in the current study. Furthermore, the complexity of neurovascular coupling and the polarity of fMRI signal in the striatum (Cerri et al. 2024) complicate interpretation. In awake rats, fMRI signal decreases were observed in several cortical, thalamic and hypothalamic regions, potentially reflecting task-induced deactivation (Binder 2012). A widespread cortical signal decrease in response to whisker pad stimulation has also been reported earlier (De Celis Alonso et al. 2008). Interestingly, the negative responses in the current work developed several seconds after the stimulus onset and then stabilized, persisting throughout the rest of the stimulation period. Considering this alongside the widespread positive responses, we speculate that the negative responses may also reflect a slow, large-scale reallocation of blood flow during prolonged stimulation, a phenomenon well-known at more local levels (Harel et al. 2002). Regardless, the temporal evolution of the negative signals does not indicate an artefactual origin.

### Stimulation frequency-dependent modulation and temporal characteristics of regional signals

Few fMRI studies have explored the signal changes to whisker pad stimulation in the primary sensory areas of rats with different stimulation frequencies. Under anesthesia, increasing response amplitudes in S1bf have been reported up to 5 Hz (range of 1 to 10 Hz) (De Celis Alonso et al. 2008), 5 Hz (range of 1 to 20 Hz) (Devonshire et al. 2012), or 12 Hz (range of 4 to 30 Hz) (Sanganahalli et al. 2008), with reduced or saturated response strengths at higher frequencies. In awake rats, 40 Hz stimulation yielded stronger responses than 5 Hz (Martin et al. 2013a). These findings align with our results, where the highest response amplitudes in S1bf were observed after mid- and high-frequency stimulation in anesthetized and awake rats, respectively. Additionally, these findings together indicate that anesthesia alters the fMRI response profile to whisker pad stimulation, with peak response amplitudes not necessarily corresponding to the natural low-frequency whisker movement (4 to 12 Hz) as previously suggested (Sanganahalli et al. 2008; Devonshire et al. 2012). Supporting this, laser Doppler flowmetry studies found maximal cortical blood flow to electrical whisker pad stimulation with 5 Hz stimulation under anesthesia, but a linear increase in blood flow was observed in awake rats up to 40 Hz stimulation (Martin et al. 2006b). With respect to areas other than S1bf, Devonshire et al. (2012) reported saturated fMRI responses in VPM after 5 Hz, and linearly increasing fMRI responses in Sp5 up to 20 Hz under anesthesia, closely matching our results.

Perhaps the most interesting findings of the current work are related to the stimulation frequency-independent modulation and temporal characteristics of fMRI signals outside the core pathway in awake rats. While fMRI responses in the core regions followed the whisker pad stimulation frequency almost linearly, most auditory, visual, and high-order regions exhibited no or a non-linear dependence between stimulation frequency and fMRI response amplitude. We can speculate that the exact primary sensory information is thus not carried over to the cross-sensory regions, suggesting distinct processing mechanisms for the primary and cross-sensory information types. For instance, the information reaching non-core systems may be more refined rather than raw. Whether these differences involve the specificity or encoding of sensory information remains unclear and requires further electrophysiological studies. Interestingly, the stimulation frequency-independent modulation observed in auditory, visual, and high-order regions resembled each other, which may indicate that the cross-sensory and associative processing share similar features distinct from the processing of detailed primary sensory information.

In addition to the distinct stimulation frequency-dependent response profiles, we observed different temporal signal shapes between core and non-core regions. The key nodes of whisker-mediated tactile system exhibited a sustained signal increase throughout the stimulation period, while the signal in non-core cerebral regions declined to a lower level shortly after the initial peak. This may indicate that adaptation to repetitive stimulus in cross-sensory and associative processing occurs more quickly than in the primary system, i.e. the cross-sensory processing may be more sensitive to changes rather than to the actual sensory input. These differing temporal profiles may even hinder the detection of such activity using conventional analysis approaches, potentially explaining the lack of clear responses in the anterior cingulate area observed in this study. The fMRI signal changes in the cross-sensory regions are likely explained by increasing neuronal firing rates, which have been observed in the cross-sensory thalamic and cortical neurons (Sieben et al. 2013). However, the evoked cross-sensory activity has also been associated with the phase reset of β-γ band oscillations in non-core cortical networks (Sieben et al. 2013). A sudden, synchronized firing of a large neuron population may create a high, acute demands on energy metabolism, potentially manifesting a hemodynamic signal.

As described earlier, the cerebellum exhibits several layers of activity that may be difficult to interpret from hemodynamic signals. H7a, H7p, and V7–8 exhibited linearly increasing fMRI responses with elevations in the stimulation frequency, suggesting that input from the primary sensory system at least partially drives the underlying neuronal activity in these regions. As H7a and H7p receive inputs from the whisker pad (Shambes et al. 1978; Bosman et al. 2010; Tsutsumi et al. 2023) and V7–8 from the primary sensory cortices (Suzuki et al. 2012), both pathways appear relevant. Despite showing similarities in the stimulation frequency-induced modulation compared to the primary pathway, the early signal decay in the cerebellum may hint at the presence of faster adaptation mechanisms to repetitive stimulus. The absence of the second peak in the response shape may be due to several factors, such as a lack of sustained neuronal activity, different processing strategies, or distinct mechanisms of functional hyperemia between the cerebellum and cerebrum.

### The impact of anesthesia on cross-sensory and high-order processing

Comparisons between awake and sedated states in fMRI have typically focused on functional connectivity or responses in primary cortical areas to sensory stimuli. However, no fMRI study has yet comprehensively characterized or compared cross-sensory, brain-wide activity between awake and anesthetized conditions. fMRI responses to visual stimulation have been reported in cortical auditory areas in both alert and anesthetized monkeys, being larger in the alert condition (Kayser et al. 2007). Similarly, we observed cross-sensory signal changes to whisker pad stimulation in Aud in both anesthetized and awake rats, with the responses being stronger in awake animals. Unlike previous reports, however, our whole-brain approach enabled a comprehensive evaluation of how anesthesia affects cross-sensory responses.

Overall, the results in awake and anesthetized animals exhibited many similarities. In both conditions, whisker pad stimulation elicited responses in the key nodes of the whisker-mediated tactile system, certain parts of auditory and visual tracts, and cerebellar regions receiving inputs from the whisker pad. These regions likely form the core structure for the low-level sensory processing of whisker-mediated tactile information, where cognition, attention, and decision-making play minimal roles. Notably, the signal changes in Pr5/Sp5 were similar under both conditions, suggesting that sensory input is perceived at the lowest level with little to no influence from the Iso + Med anesthesia.

Nevertheless, there were multiple observations indicating that anesthesia masked brain-wide responses to sensory stimulation. First, in the anesthetized animals, many regions showed no evoked activity. Second, similarly to previous observations (Kayser et al. 2007), anesthesia restricted fMRI responses to a smaller area compared to conscious rats. Third, anesthesia distorted the linear stimulation frequency-dependent behavior of fMRI responses along the primary sensory tract. Fourth, the lack of linearity in the fMRI responses under anesthesia led to averaged response profiles that lacked detail, preventing physiologically relevant clusterization. Fifth, this lack of details also applied to the temporal signal characteristics, hindering relevant clusterization. Therefore, based on the anesthesia data alone, it would not have been possible to conclude that the core and non-core regions express distinct stimulation frequency-dependent modulation and temporal characteristics of fMRI signals.

As anesthesia is considered to disrupt integrative processes (Montupil et al. 2023), the absence of fMRI responses in the high-order regions (RSC, PHC, and TeA) is not surprising. If the cross-sensory information flow depends on the feedback projection from integrative regions or cortico-cortical projections, it may explain the smaller evoked cross-sensory activity in Aud and Vis under anesthesia. However, PPC responded to electrical whisker pad stimulation in the anesthetized rats, as also observed in mice (Dinh et al. 2024), which indicates that certain associative regions can still respond to strong stimulation despite anesthesia. Interestingly, V7–8 was the only cerebellar region showing no significant signal changes to stimulation in the anesthetized animals. As V7–8 likely receives whisker-mediated sensory inputs from the cerebral cortex (Suzuki et al. 2012), our observation suggests that cortico-cerebellar circuits or prior integrative processes may be suppressed by anesthesia. Alternatively, the signal changes in V7–8 may be related to a coordination of muscle activity (Manni and Petrosini 2004) during the awake state. Despite being weak in amplitude, the responses in H6, H7a, and H7p appeared robust under anesthesia, consistent with previous reports of neuronal responses in H7a to electrical whisker pad stimulation in both awake and anesthetized mice (Bosman et al. 2010). Similarly, robust cerebellar responses to other sensory stimuli, such as auditory (Blazquez Freches et al. 2018) and hind−/forepaw (Peeters et al. 1999), have been observed under anesthesia, further supporting the reliability of cerebellar mapping in unconscious rodents.

### Limitations

Neurovascular coupling may vary along the whisker-mediated tactile pathway (Devonshire et al. 2012). Nevertheless, we assume that the region-specific stimulation frequency-modulated change in the fMRI signal represents a qualitative change of the underlying neuronal activity. Moreover, assuming similar neurovascular coupling within a given larger region, we can functionally differentiate, e.g. two cortical structures based on their signal behavior.

Several measures were implemented to minimize the effects of movement on the data: (i) animals were carefully habituated for the imaging procedure; (ii) a quiet and movement-tolerant zero-TE sequence, previously demonstrated to perform excellently in awake fMRI studies, was used; (iii) robust surgical head-fixation was applied; (iv) data were motion corrected using standard tools; and (v) motion correction parameters were included as regressors in statistical map and time series analyses. The raw data contained minimal movement, and temporal ROI signal profiles indicated hemodynamic origins without notable spike-like artifacts. Therefore, although movement-induced confounds cannot be completely ruled out, they seem unlikely.

The mechanical air-puff stimulation introduced a sound source overlapping with the stimulus paradigm. To minimize its effect, ear plugs were used in both anesthetized and awake rats, and the air pressure was kept as low as practically feasible. Furthermore, experiments with mechanical stimulation were partially controlled by using inaudible electrical stimulation. Indeed, we observed activation of the auditory tract in response to both electrical and mechanical stimuli. While awake mice have exhibited bilateral auditory responses to a noisy unilateral mechanical whisker pad stimulation apparatus (Chen et al. 2020), only unilateral auditory activity was observed in the current study. Nevertheless, we cannot fully exclude the potential confounding effect of audible noise on the data obtained with mechanical stimulation.

Another limitation is the repeated use of isoflurane. Since even single exposure to surgical-level isoflurane anesthesia can affect the brain for weeks (Stenroos et al. 2021), isoflurane may have induced unknown long-term effects on cerebral function.

In addition to the inherent spatial and temporal inaccuracy of hemodynamic responses, the isotropic spatial resolution of 625 μm in our functional images poses certain limitations. Because of the resolution, the analyses were restricted to larger anatomical areas, excluding potentially relevant areas at the scale of a few voxels. Additionally, only small parts of the large anatomical regions may contribute to multimodal activity (Driver and Noesselt 2008), which weakens our ROI-based signals when large ROIs are used. However, detailed information on intra-regional multimodal zones in the rat brain is not available yet.

Also, the 60-s inter-stimulus interval appeared too short for signals to reach baseline in some ROIs during awake experiments. Therefore, longer intervals are recommended for similar future studies.

Lastly, a few factors may explain the generally weak amplitudes of the fMRI responses in this study. First, differences in the origin of functional contrast between blood oxygenation level dependent measurements and zero-TE fMRI methods (Lehto et al. 2017) can lead to different absolute response amplitudes but similar functional contrast-to-noise ratios (Paasonen et al. 2022). Second, whisker pad stimulation may be 1 of the weaker physiological stimuli used in preclinical fMRI, leading to signal changes as low as 0.3% even using conventional fMRI techniques (de Celis Alonso et al. 2012).

## Supplementary Material

Supplementary_materials_bhaf194

Supplementary_materials_video_1_bhaf194

Supplementary_video_2_bhaf194

## Data Availability

The data package is available at https://doi.org/10.23729/fd-b3b684c4-1a98-37fd-858a-4deba1bc799b.
