# Supplementary material for: Whisker pad stimulation with different frequencies reveals non-uniform modulation of functional MRI signal across sensory systems in awake rats
Source: Cereb Cortex. 2025 Jul 23;35(7):bhaf194. doi: 10.1093/cercor/bhaf194 (PMC12284883; doi:10.1093/cercor/bhaf194)
Supplement: Supplementary_materials_bhaf194 [file supplementary_materials_bhaf194.docx]

**Supplementary materials**

**Whisker pad stimulation with different frequencies reveals non-uniform modulation of functional MRI signal across sensory systems in awake rats**

Jaakko Paasonen^a,#,*^, Juha S. Valjakka^a,b,#^, Raimo A. Salo^a^, Ekaterina Paasonen^a,c^, Heikki Tanila^a^, Shalom Michaeli^b^, Silvia Mangia^b^, Olli Gröhn^a^

^a^ A. I. Virtanen Institute for Molecular Sciences, University of Eastern Finland, Kuopio, Finland
^b^ Center for Magnetic Resonance Research, University of Minnesota, Minneapolis, USA
^c^ NeuroCenter, Kuopio University Hospital, Kuopio, Finland

^#^Equal contribution

*Corresponding author. A.I.V. Institute for Molecular Sciences, University of Eastern Finland, P.O. Box 1627, FI-70211, Kuopio, Finland. Phone number: +358403552219. E-mail address: [jaakko.paasonen@uef.fi](mailto:jaakko.paasonen@uef.fi)


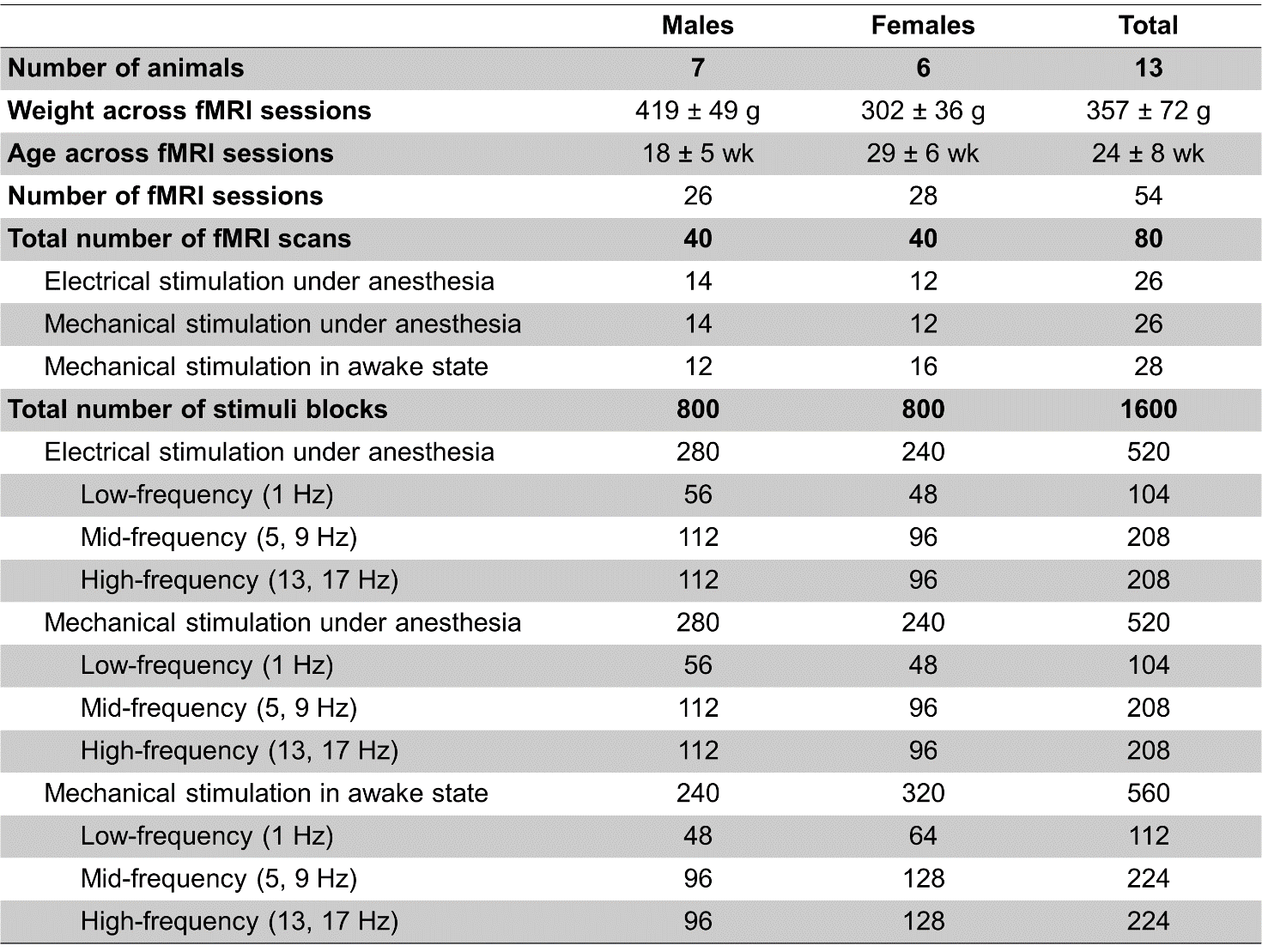


**Supplementary Table 1.** Numerical details related to the animals and stimulation experiments. fMRI, functional magnetic resonance imaging.


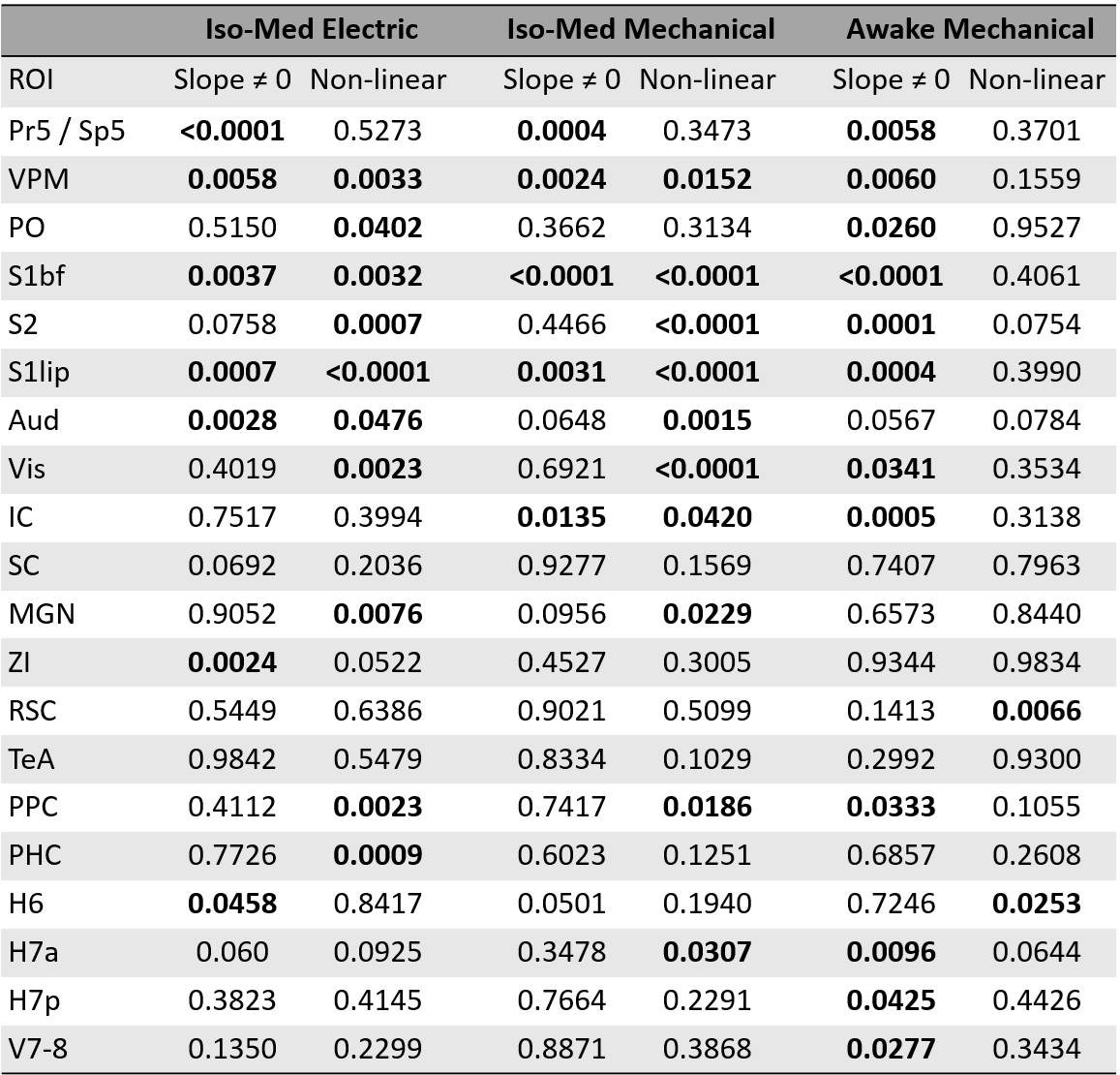


**Supplementary Table 2. The obtained uncorrected p-values for estimating the significance of the slope and the linearity between the average functional magnetic resonance imaging response and the stimulation frequency.** For each region-of-interest and group, we tested whether the slope between stimulation frequency and average response deviated from 0 (t-test), and whether the relationship between the stimulation frequency and average response was linear (normality test for residuals of the fit). Iso+Med, isoflurane and medetomidine anesthesia.


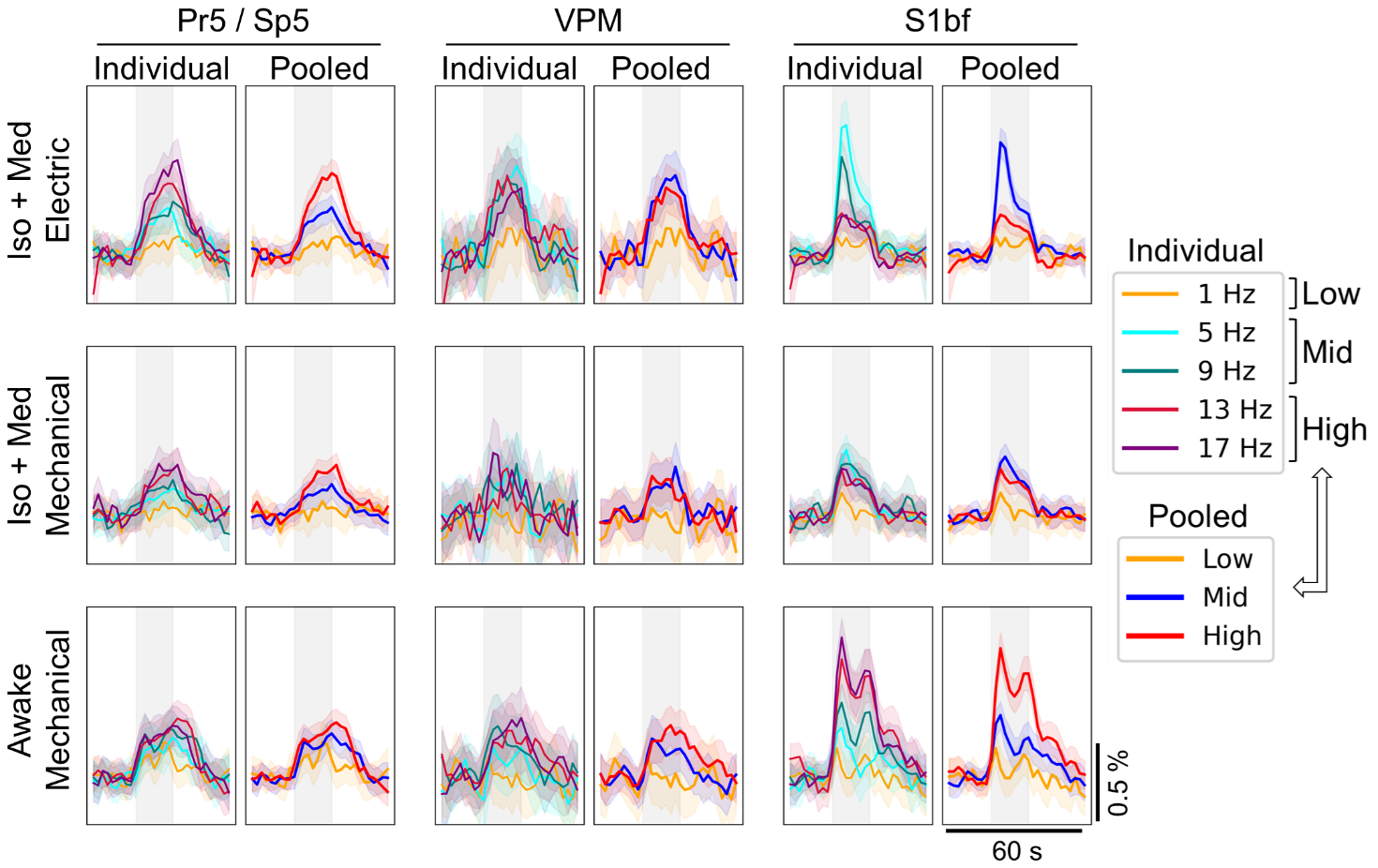


**Supplementary Figure 1. Frequency-specific and pooled time series obtained from the key nodes of the whisker-mediated tactile system.** Because of the similarity of the responses to certain stimulation frequencies, their correspondence to natural whisking frequencies, and to simplify the representation of complex and multilayered data, the five different stimulation frequencies were pooled into three subgroups. Each frequency-specific and pooled mean time serie consists of 104-112 or 208-224 repetitions, respectively. The shaded vertical gray region indicates the timing for the 16-s stimulus block. The 90% confidence interval is shown as a shaded region around the mean time series. Pr5/Sp5, principal and spinal trigeminal nuclei; S1bf, primary somatosensory cortex barrel field region; VPM, ventral posteromedial thalamic nuclei.


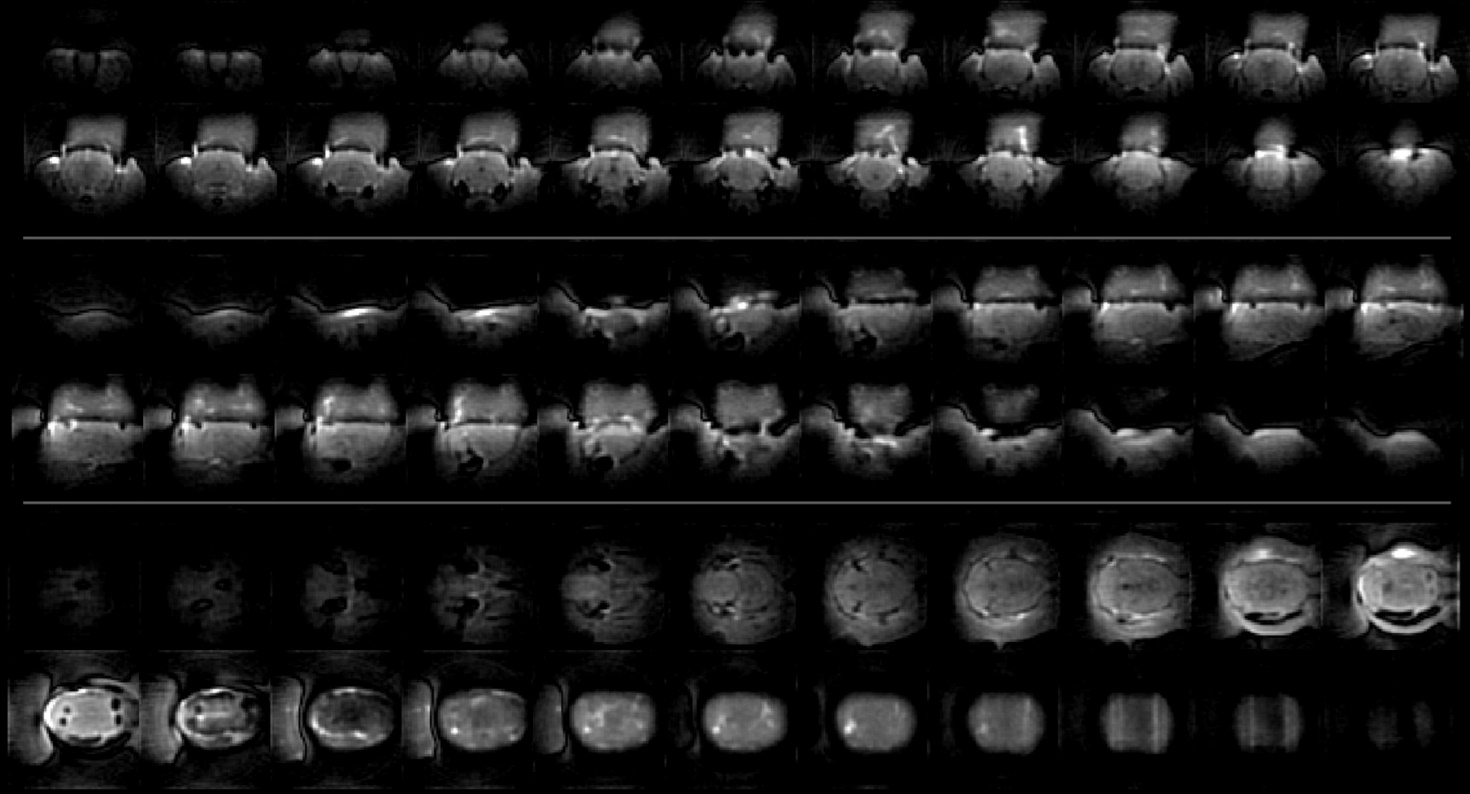


**Supplementary Figure 2. Raw fMRI images obtained from a representative rat.** One 3D volume consists of 64 slices in all directions, with an isotropic resolution of 625 µm and whole-brain coverage. 22 representative slices, selected at 1250 µm intervals (every other slice), are shown for each orientation (coronal, sagittal, and axial). The four black signal voids located above the olfactory bulb and cerebellum (visible in bottom-left image) are caused by relatively big anchoring screws used for the head-implant. The signal visible on top of the skull originates from the head-implant.


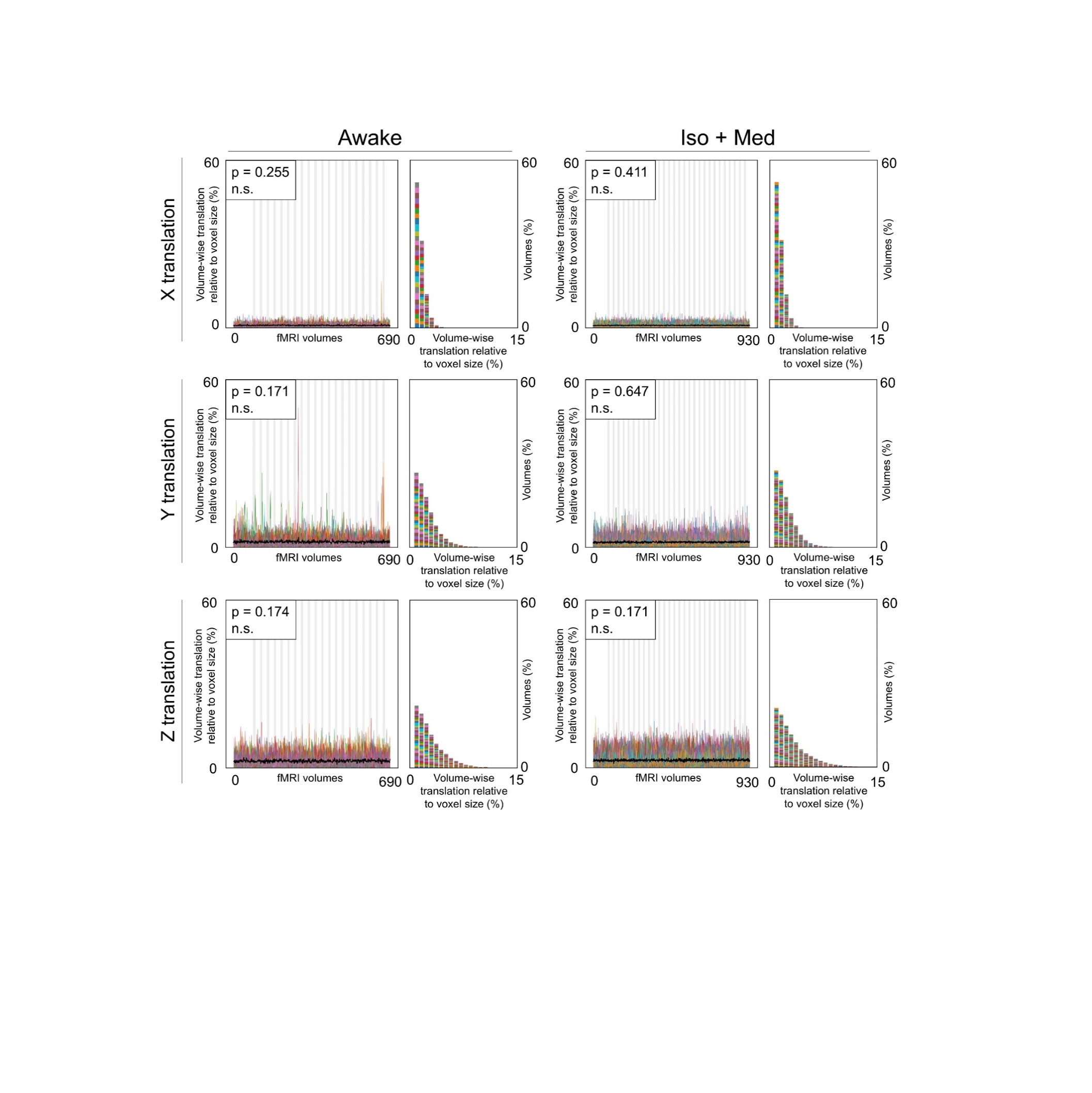


**Supplementary Figure 3. Summary of X, Y, and Z translation parameters obtained from the motion correction.** Time series for each translation parameter in each fMRI scan (Awake, n = 26; Iso+Med, n= 52) are shown on the left side of each subplot. Each point in the time series represents the absolute volume-wise translation value. The thick black line indicates the average value across volumes. X and Z translation values were similar between awake and anesthetized rats. A few awake scans showed Y-axis translations of up to a half voxel. There was no significant increase in translation during the stimulus period (shaded vertical gray region), as determined by a two-sample t-test (uncorrected p > 0.171 in all cases). The distribution of volume-wise translation values for each fMRI scan is shown on the right side of each subplot. The order and color of the data sets are consistent across bars. The Y-axis represents the total number of volumes across all scans. The vast majority of volumes acquired during both awake and anesthetized experiments exhibited translation values less than 5 % of the voxel size.


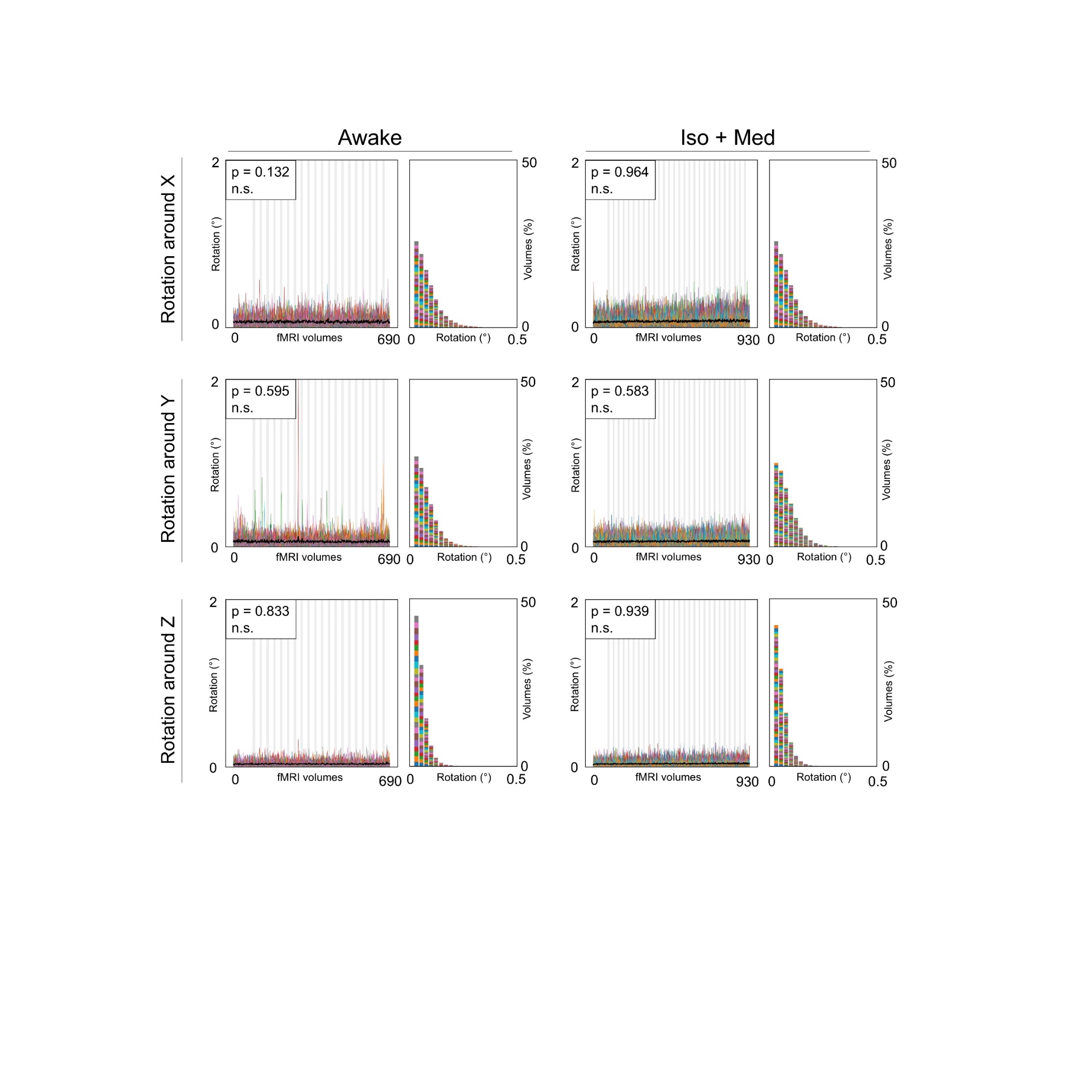


**Supplementary Figure 4. The summary of rotation parameters around X, Y, and Z obtained from the motion correction.** Time series for each rotation parameter in each fMRI scan (Awake, n = 26; Iso+Med, n= 52) are shown on the left side of each subplot. Each point in the time series represents the absolute volume-wise rotation. The thick black line indicates the average rotation value across volumes. Rotations around X and Z were similar between awake and anesthetized rats. A few awake scans showed rotation around Y-axis of up to 2°. There was no significant increase in rotation values during the stimulus period (shaded vertical gray region), as determined by a two-sample t-test (uncorrected p > 0.132 in all cases). The distribution of volume-wise rotation values for each fMRI scan is shown on the right side of each subplot. The order and color of the data sets are consistent across bars. The Y-axis represents the total number of volumes across all scans. The vast majority of volumes acquired during both awake and anesthetized experiments exhibited rotation values less than 0.2°.


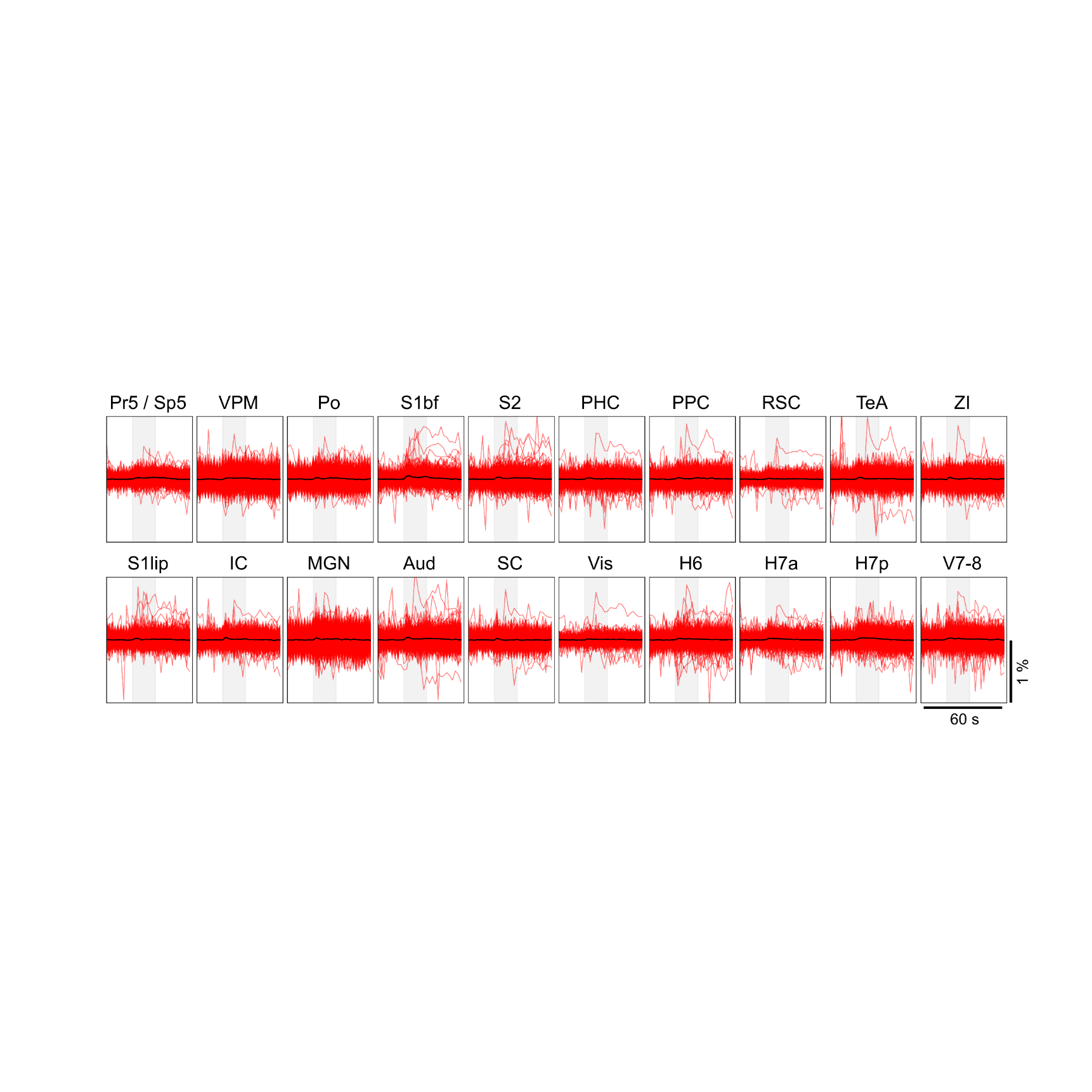


**Supplementary Figure 5. Individual region-specific time series from the awake experiments.** Each plot displays 560 region-specific time series (red lines), aggregated across all stimulation frequencies. The shaded vertical gray region indicates the 16-s stimulus block. Importantly, only a few of the 560 time series exhibit distinct amplitude changes, suggesting the absence of prominent large-scale spike-like artifacts in the fMRI signals that could result from movement. The average responses (thick black line) appear small, as frequencies eliciting no significant responses are also included.


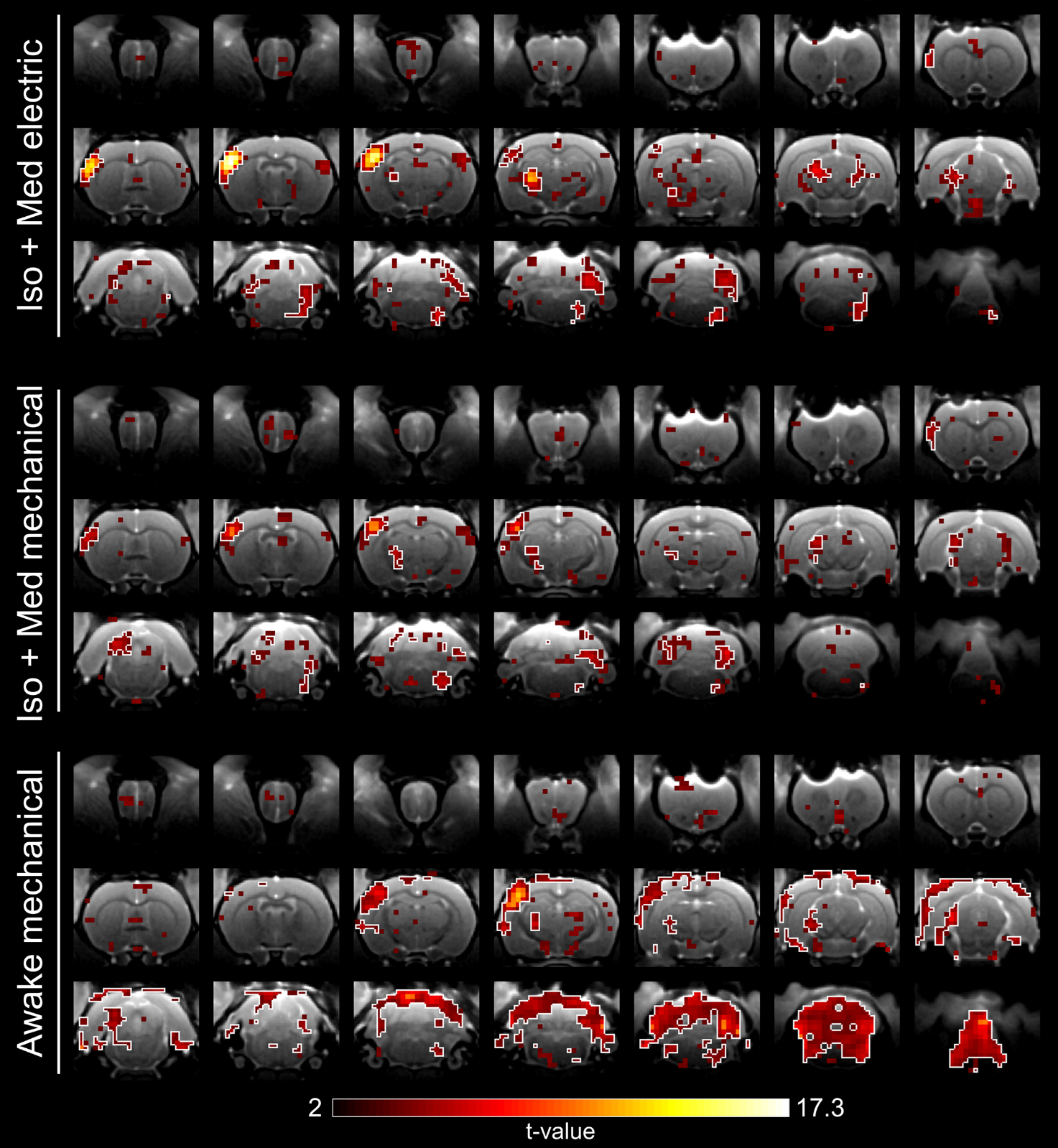


**Supplementary Figure 6. Group-level statistical** **maps in response to mid-frequency whisker pad stimuli, shown across 22 representative slices.**  White outlines on the statistical maps indicate regions with p < 0.005 (FWE-corrected), while the color maps are thresholded at t > 2. Results are based on 208-224 stimulus blocks collected from 26-28 scans in each group. Statistical maps are overlaid on high-resolution anatomical images. Slices are displayed at 1250 µm intervals (625 µm slice thickness, i.e. every other slice).


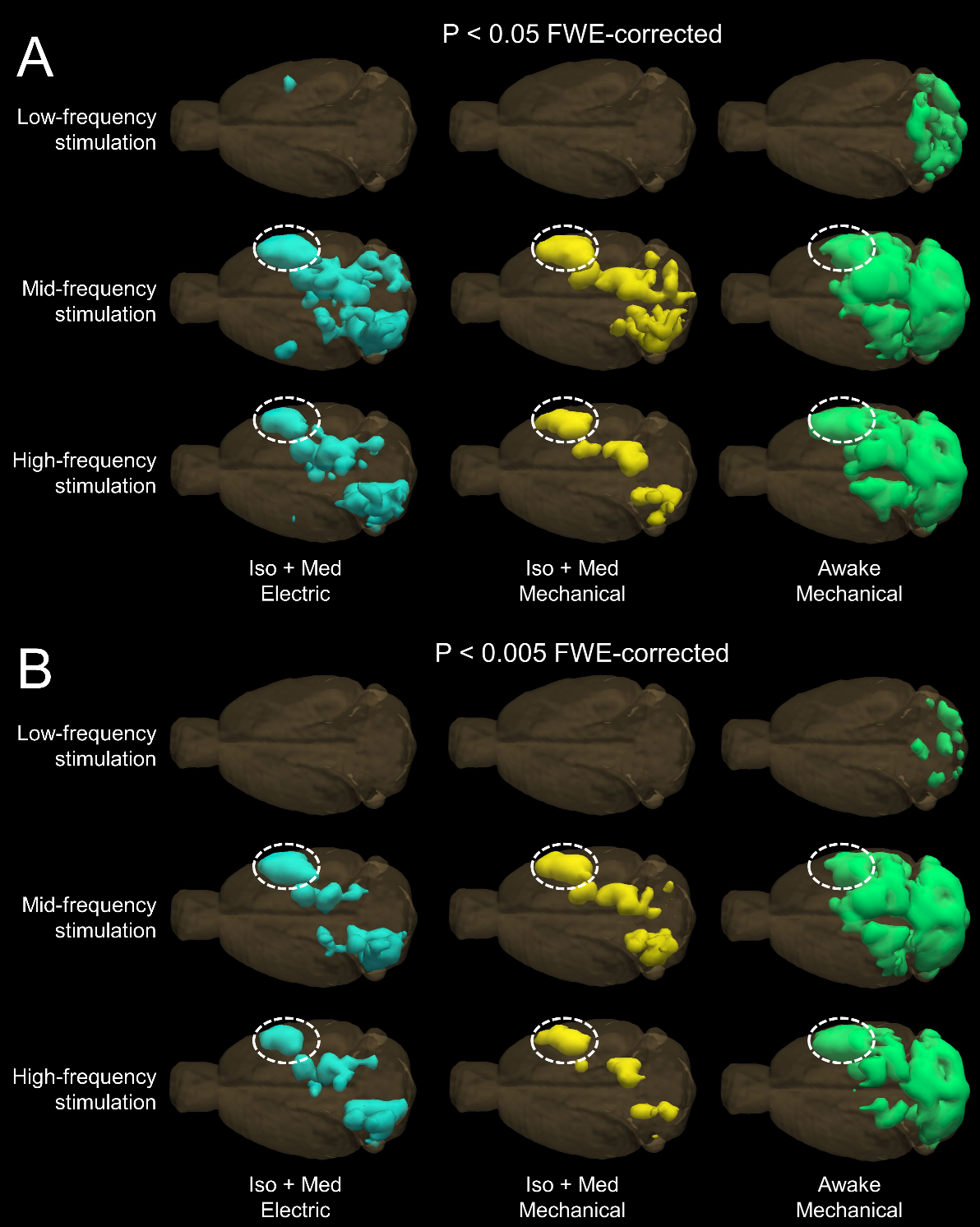


**Supplementary Figure 7. 3-D illustration of significant voxels (A, p<0.05, FWE-corrected; B, p<0.005, FWE-corrected) obtained with low-, mid-, and high-frequency stimuli in each group.** The results are obtained from 104-224 stimuli blocks per map. Generally, low-frequency stimuli produced the spatially smallest responses, and the localization of signal changes to mid- and high-frequency stimuli resembled each other. However, there were also clear differences in the statistical maps to the mid- and high-frequency stimuli. As an example, the high-frequency stimulus yielded spatially more widespread signal changes within the S1BF (dashed ellipsoid) in awake rats [83 vs 63 significant voxels (p<0.005, FWE-corrected)], but more restricted signal changes in anesthetized rats with both electrical [43 vs 67 significant voxels (p<0.005, FWE-corrected)] and mechanical [50 vs 70 significant voxels (p<0.005, FWE-corrected)] stimuli. These findings suggest that anesthesia can affect the relationship between stimulation frequency and the spatial extent of the fMRI responses within regions. Iso+Med, isoflurane and medetomidine anesthesia.


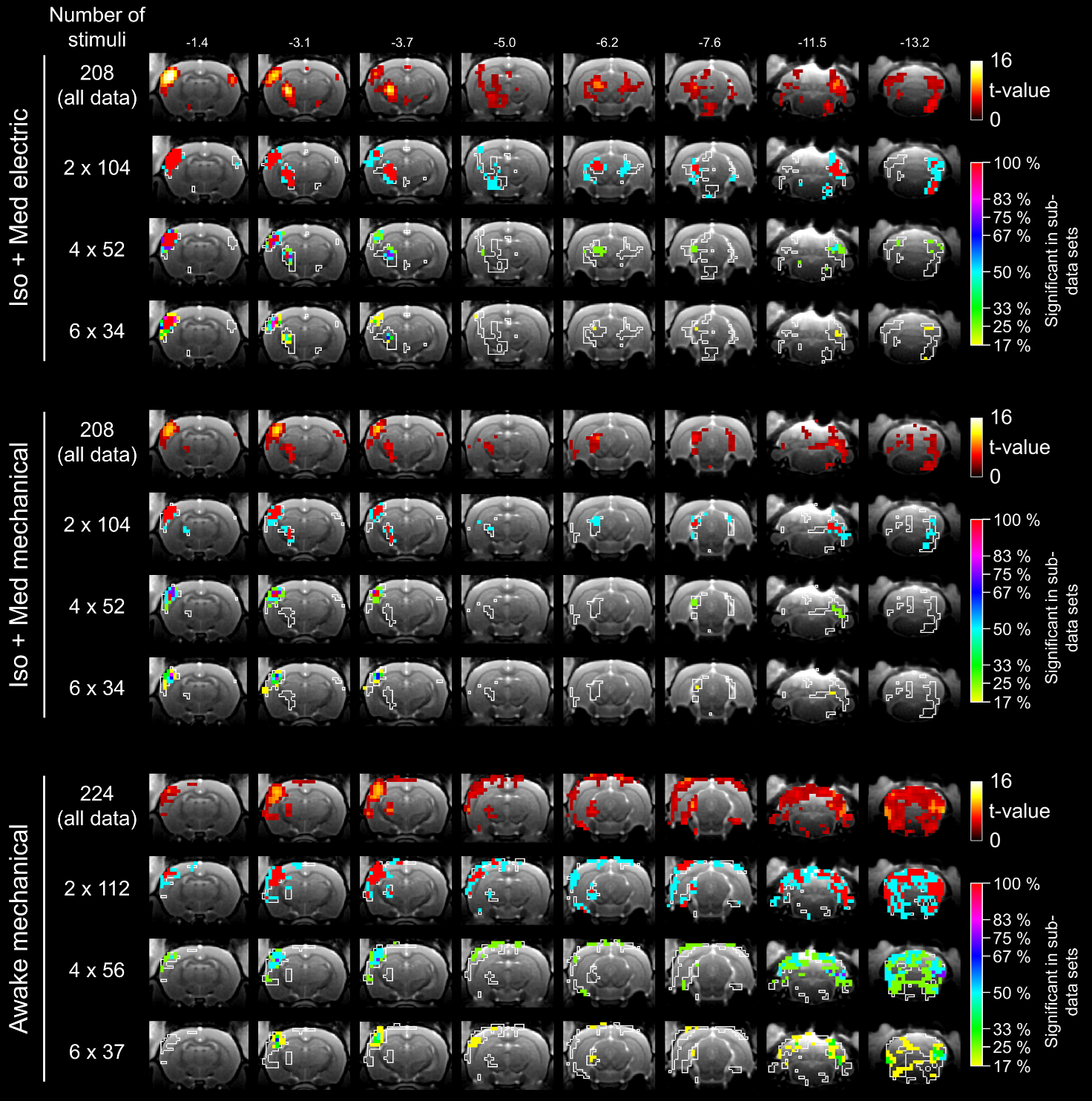


**Supplementary Figure 8. The effect of data quantity on statistical maps for mid-frequency whisker pad stimulation.** The top row in each group shows significant (p < 0.005, FWE-corrected) voxels obtained with the full dataset (208-224 stimuli). White outlines on the maps in the subsequent rows correspond to the significant regions from the top row, enabling direct comparison of how reducing the amount of data restricts the activation pattern. These rows display summary maps generated by dividing the full dataset into two (104-112 stimuli), four (52-56), or six (34-37) equally-sized sub-datasets. The color-coding in these maps indicates the incidence of significant (p < 0.005, FWE-corrected) voxels across the sub-datasets, which can be interpreted to resemble the likelihood of detecting significant activity using a dataset of that size. The values above the top row indicate the approximate distance from bregma for each slice. Statistical maps are overlaid on high-resolution anatomical images.


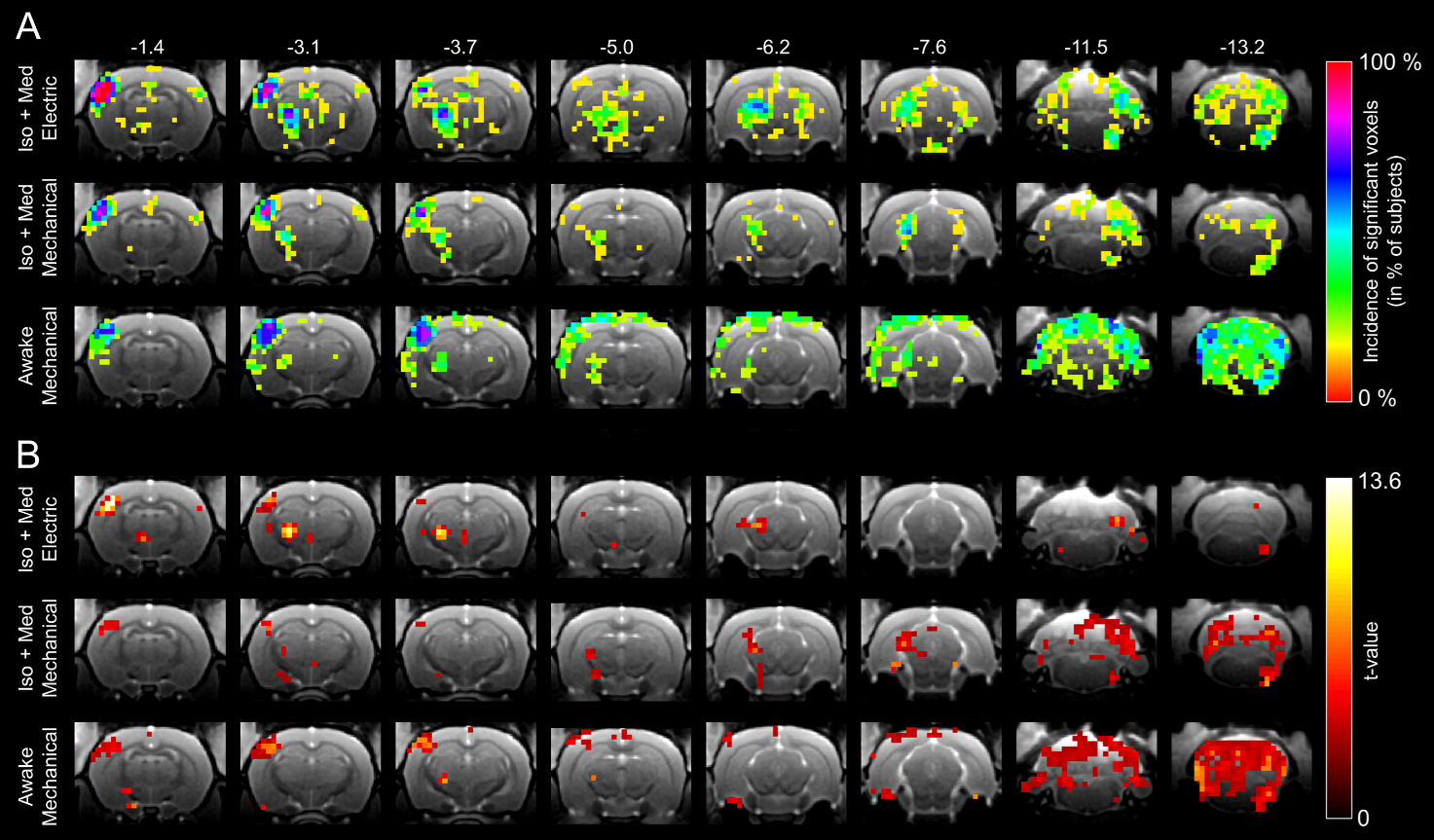


**Supplementary Figure 9. Summary of individual-level statistical maps (A) and statistical maps from a representative subject (B).** In panel A, the color-coding indicates the percentage of subjects (n = 13 for anesthetized and n = 10 for awake conditions, respectively) who individually expressed significant (p < 0.05, FWE-corrected) signal changes in each voxel. In panel B, each row represents the activation pattern elicited by different types of stimuli in the same subject. For simplicity, all stimuli given for the subject were included in the general linear model-based analysis for both A and B. Values above the top row indicate the approximate distance from bregma for each slice. Statistical maps are overlaid on high-resolution anatomical images.


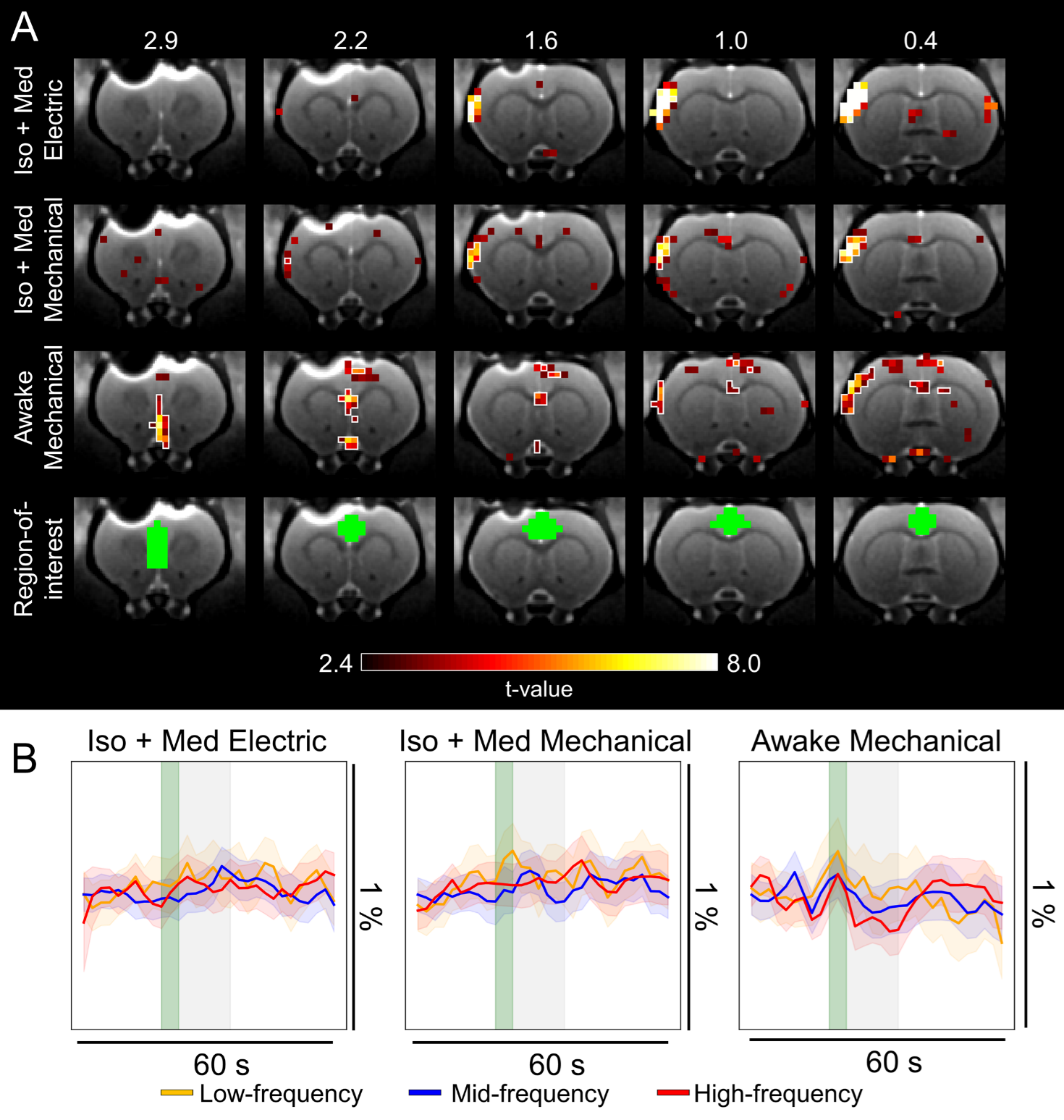


**Supplementary Figure 10. Group-level statistical maps in the frontal brain obtained with a 4-s stimulus duration (A), and time series with different frequency groups derived from the frontal cingulate area (B).** Significant voxels (white outline, p < 0.05 FWE-corrected) were detected near the anterior cingulate area in awake animals. In these animals, the positive response was brief across all stimulation frequencies (B, right panel). Color maps are thresholded at t > 2.4. The results are based on 520-560 stimulus blocks collected across 26-28 scans per each group. All stimulation frequencies were included in the generation of the statistical maps (A). Values in the top row indicate the approximate distance from bregma for each slice. Statistical maps are overlaid on high-resolution anatomical images, which include artefacts above the frontal cortex caused by relatively big anchoring screws. The shaded vertical gray region in B indicates the timing for the 16-s stimulus block. The shaded vertical green region in B indicates the timing for the 4-s analysis block. Gray and green regions overlap.


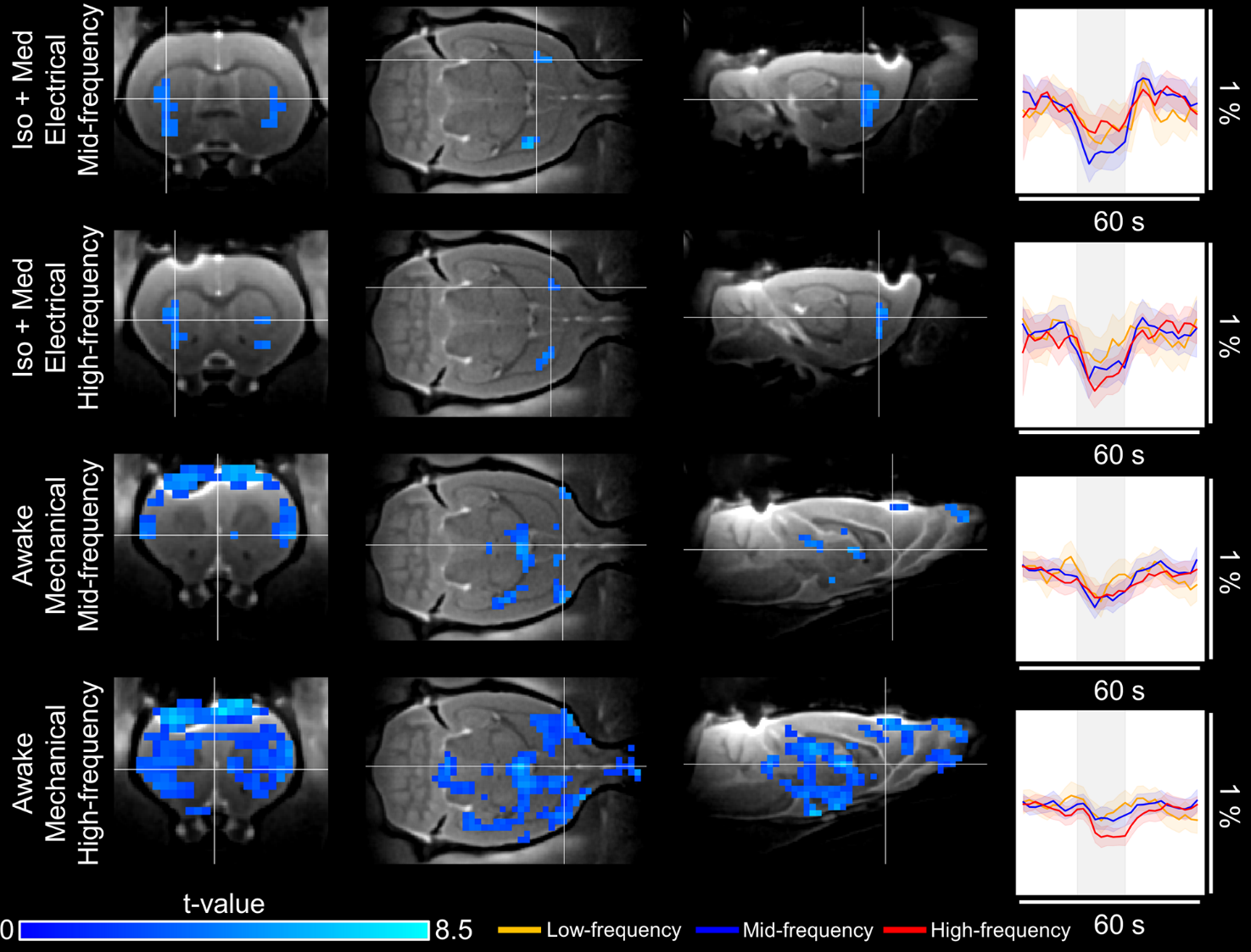


**Supplementary Figure 11. Summary of group-level statistical maps and corresponding time series showing significant (p < 0.005 FWE-corrected) negative responses to whisker pad stimulation.** The white cross indicates the slice location for each orientation. The time series on the right were extracted from a region-of-interest encompassing all condition-specific significant voxels. With electrical stimulation, signal decrease in the striatum was immediate, possibly reflecting change in neural activity. In awake experiments, the more widespread signal decrease was delayed, particularly with high-frequency stimulation, which might suggest a different underlying mechanism than an immediate response to whisker pad stimulation. No significant negative responses were detected with low-frequency stimulation in any experimental group, or with mechanical stimulation under anesthesia. Statistical maps are overlaid on high-resolution anatomical images, which include artefacts above the frontal cortex caused by relatively big anchoring screws.


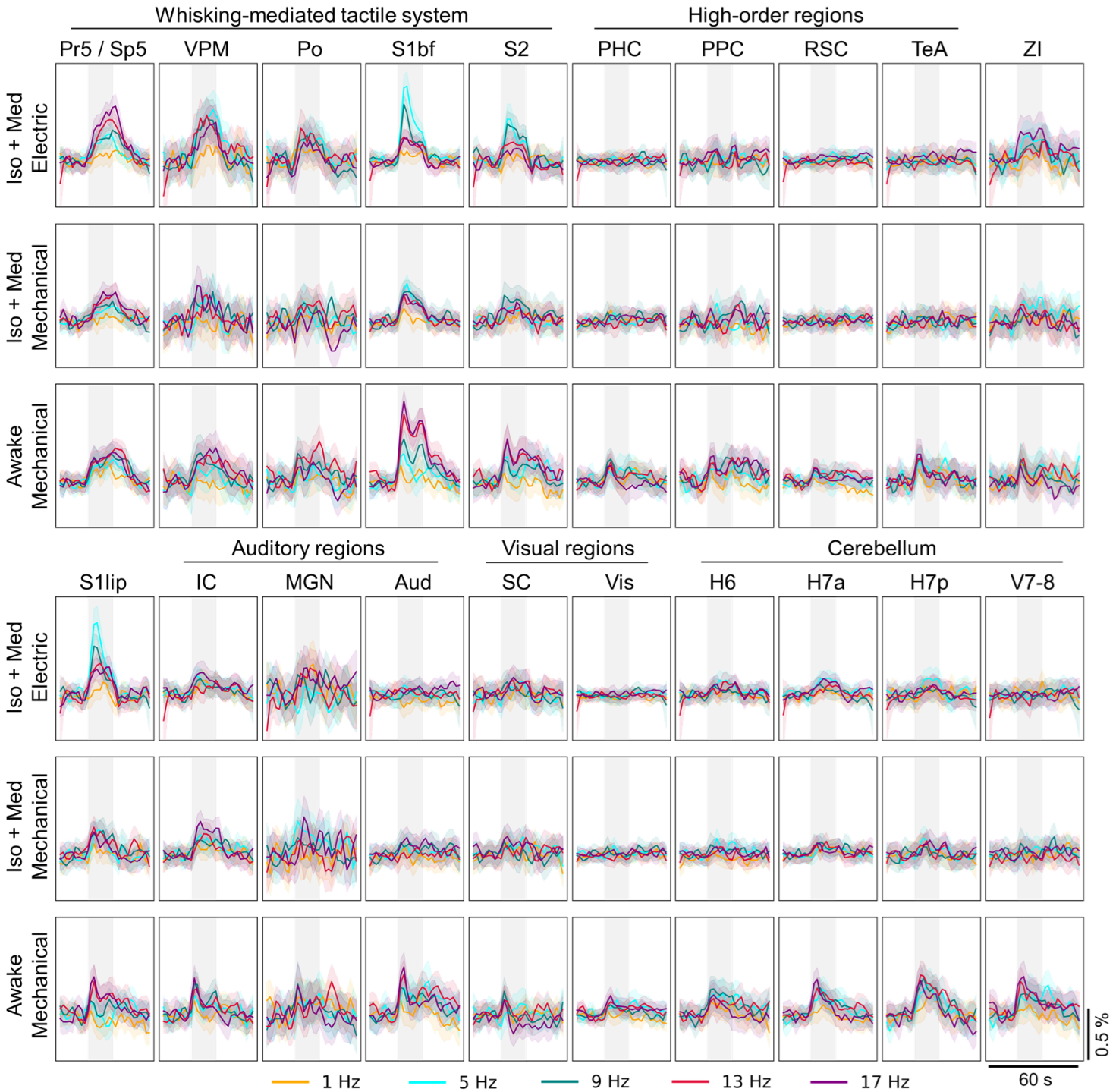


**Supplementary Figure 12. Group-level time series for each region-of-interest and stimulation frequency.** In most regions, the responses to 5 Hz and 9 Hz, and 13 Hz and 17 Hz resembled each other, which allowed us to simplify the presentation of the data into three groups. Each average time series is a result of 104-112 stimuli. The shaded vertical gray region indicates the 16-s stimulation period. Abbreviations for regions-of-interest are provided in Figure 1 and in Table 1. The 90% confidence interval is shown as a shaded region around the mean time series. Iso+Med, isoflurane and medetomidine anesthesia.


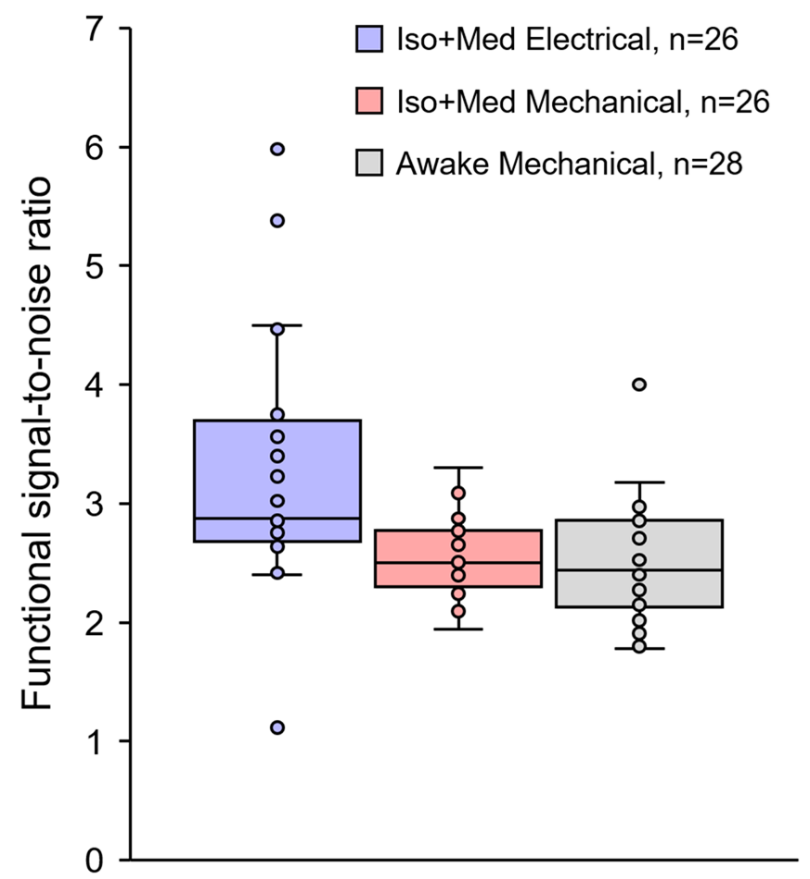


**Supplementary Figure 13. Functional signal-to-noise (SNR) ratios across all scans.** The highest SNRs were observed with electrical stimulation, while mechanical stimulation resulted in comparable but lower ratios. Signals for the calculation were extracted from the barrel field of primary somatosensory cortex (S1bf; see Figure 1). The SNR was calculated by averaging the 10 highest signal amplitudes during stimulation periods and dividing this value by the standard deviation of 10 randomly selected time points between stimulus periods. The whisker plots display quartiles, with outliers excluded. Overlapping data points are hidden, which accounts for the absence of 26 or 28 visible markers.


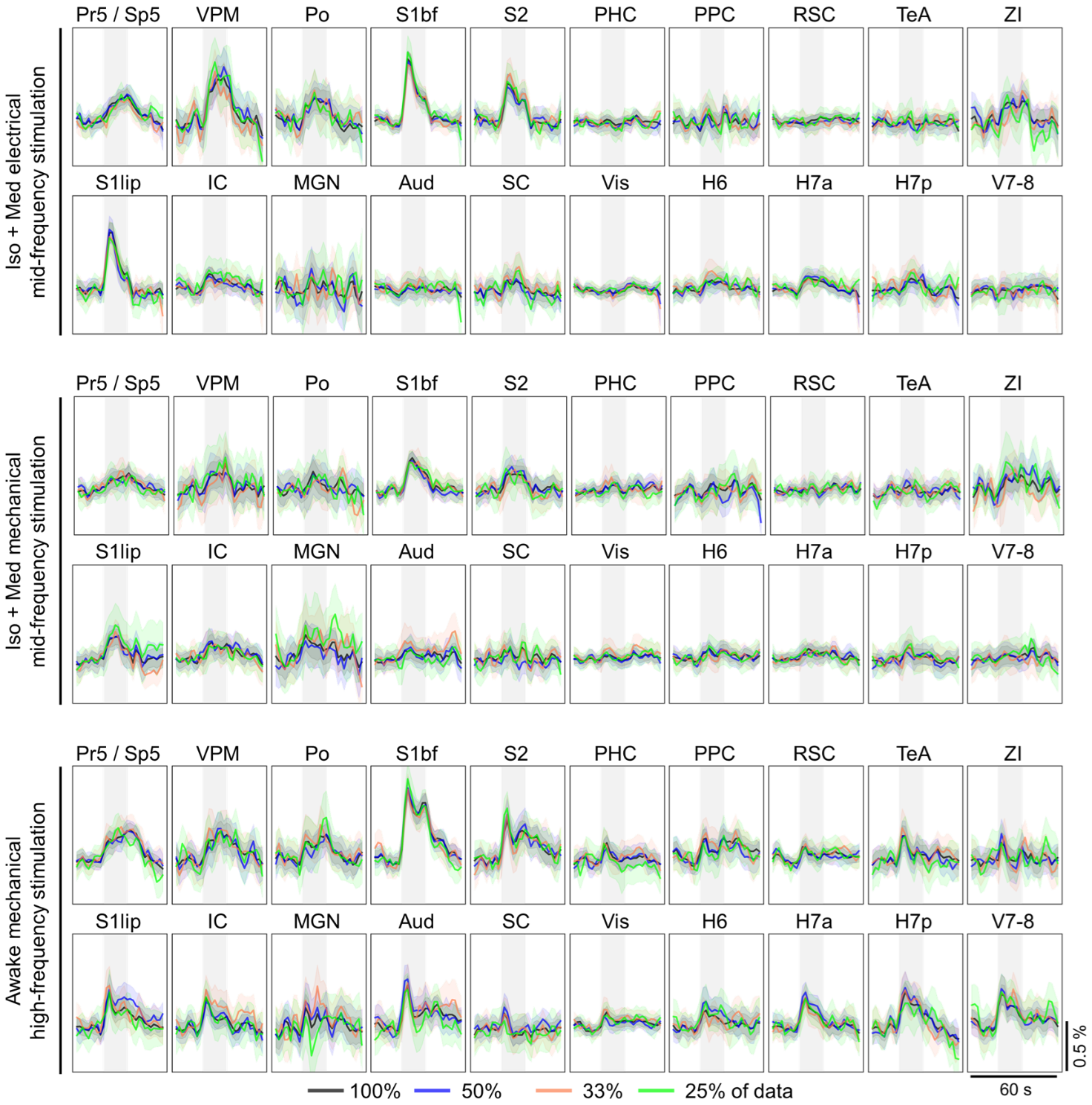


**Supplementary Figure 14. Group-level mean time series for each region-of-interest and experimental condition, shown for different sample sizes.** The original data (208-224 stimuli) are shown in black. Blue, orange, and green represent results when random subset comprising 50 %, 33 %, or 25 % of the data, respectively, were used. While reduced sample size has little effect on the average response, it increases baseline fluctuation and variability, likely reducing statistical detectability in map-based analyses. The shaded vertical gray area indicates the 16-s stimulation period. For simplicity, only one representative stimulation frequency was selected for each experimental condition. Abbreviations for regions-of-interest are provided in Figure 1 and in Table 1. The 90% confidence interval is shown as a shaded region around each mean time series.


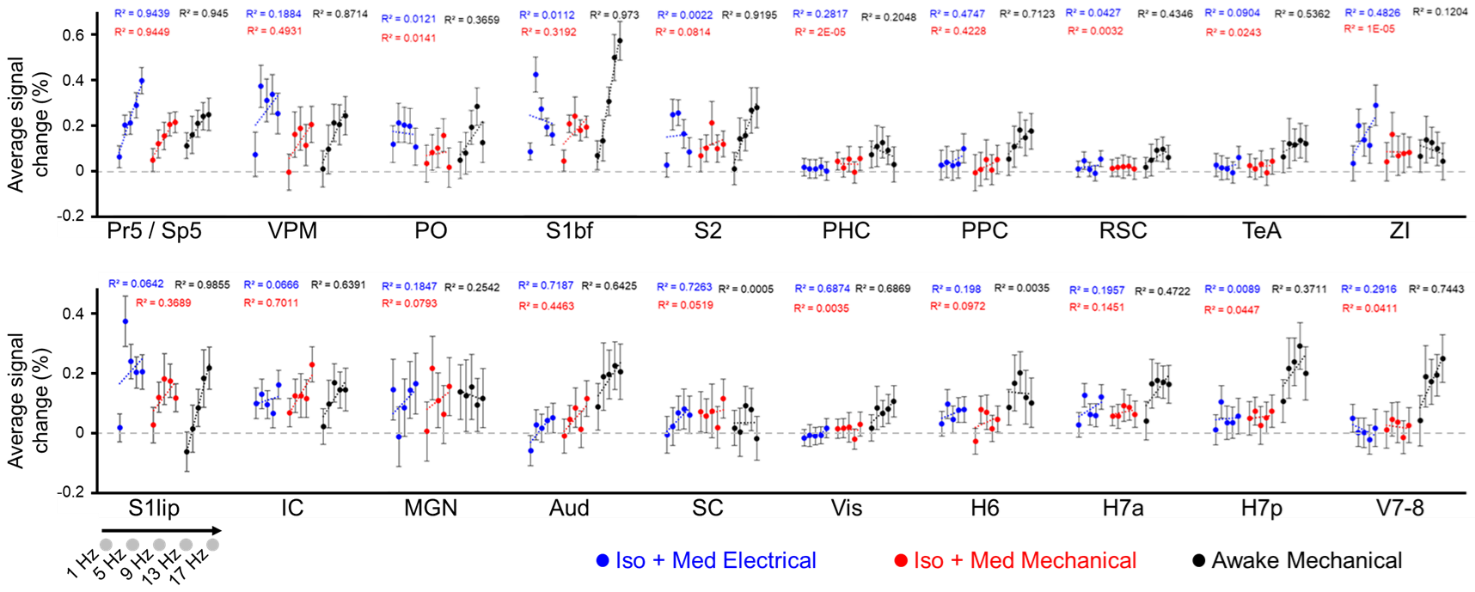


**Supplementary Figure 15. Group-level average fMRI responses for each experimental group and region-of-interest across individual stimulation frequencies.** In each subplot, the round markers correspond to stimulation frequencies 1, 5, 9, 13, and 17 Hz, in that order. The linearity of the response profiles closely resembles those shown in Figure 4. Average responses were typically calculated over a 20-s window beginning at the stimulus onset (see Materials and Methods). Error bars indicate the 90% confidence interval. Abbreviations for regions-of-interest are provided in Figure 1 and in Table 1. Iso+Med, isoflurane and medetomidine anesthesia.


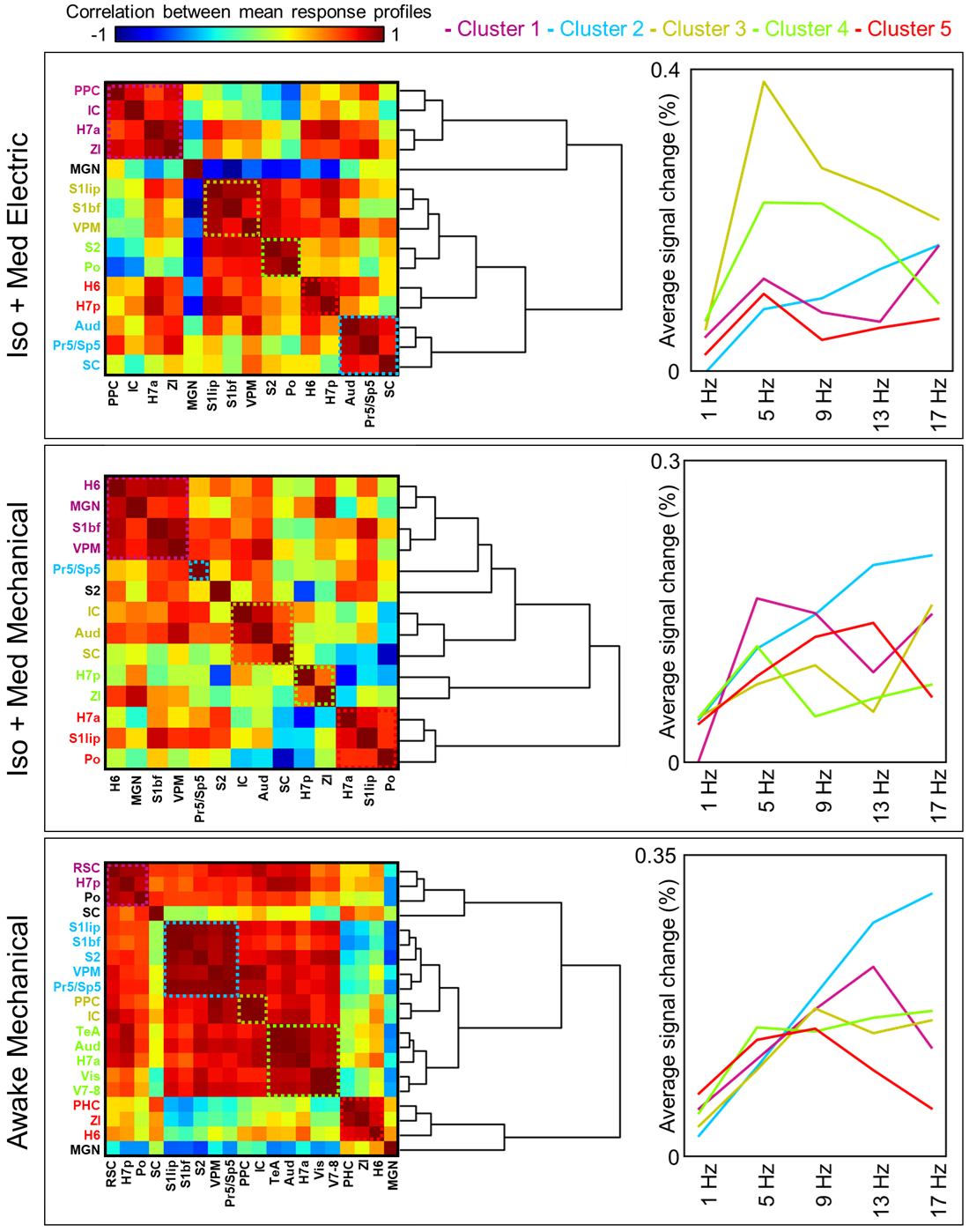


**Supplementary Figure 16. Hierarchical clustering of average fMRI response profiles using all individual stimulation frequencies.** The primary finding - the whisker-mediated tactile system clusters separately in awake but not in anesthetized animals - is consistent with the results shown in Figure 5, where three frequency groups were used. The average fMRI response profiles, derived from the data presented in Supplementary Figure 15, were analyzed using hierarchical clustering. Correlation matrices on the left indicate the similarity between frequency response profiles across regions-of-interest. The resulting hierarchical trees (dendrograms) are shown to the right of the matrices. Average response curves were then computed for the five main clusters in each group and are displayed on the far right. Clusters containing only a single region and high level of hierarchy were excluded from the illustrations. Regions without significant signal changes (Table 1) were omitted from the analysis. The cluster including brainstem nuclei is color-coded in light blue across all groups. Abbreviations for regions-of-interest are provided in Figure 1 and in Table 1. Iso+Med, isoflurane and medetomidine anesthesia.


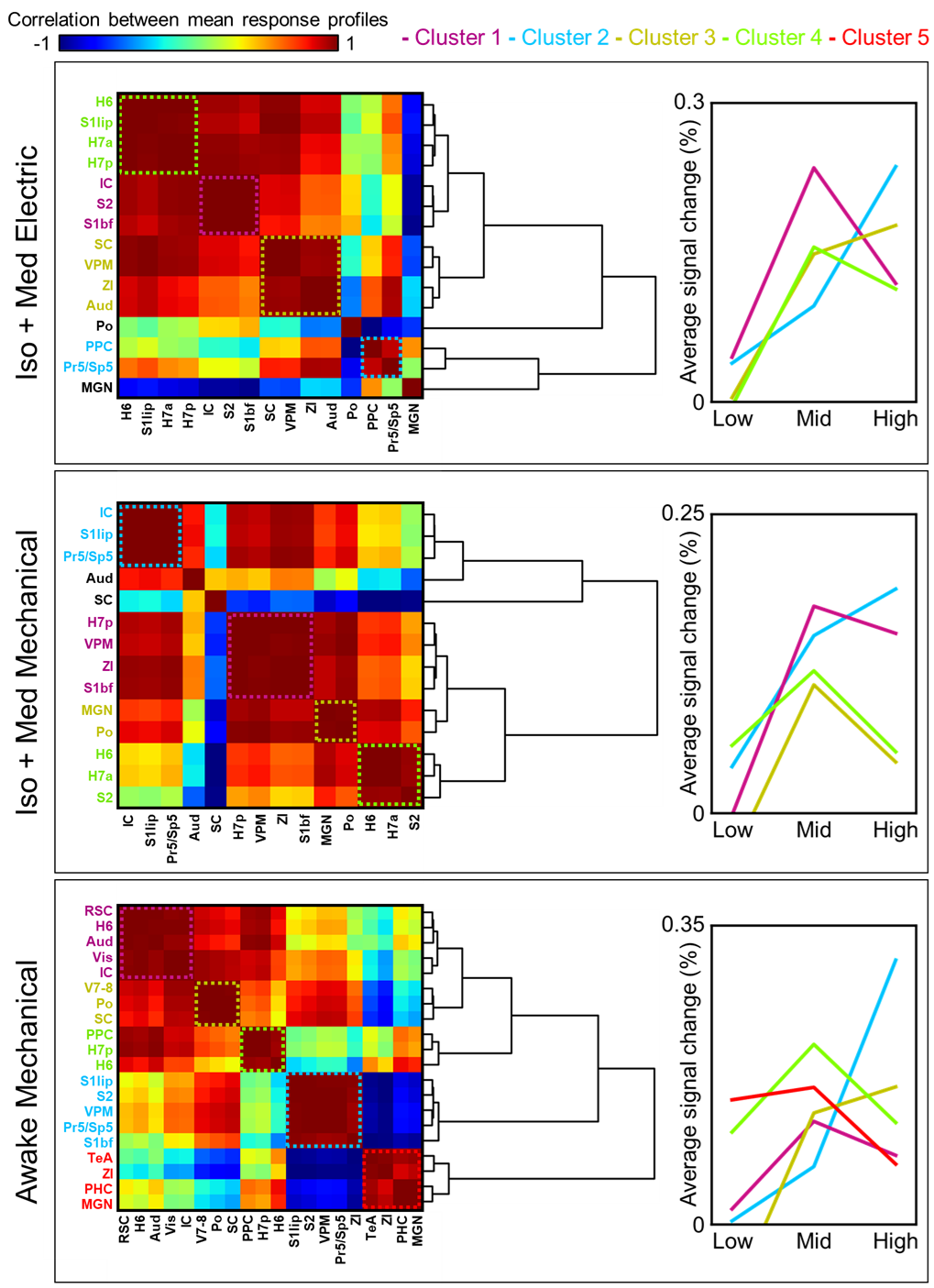


**Supplementary Figure 17. Hierarchical clustering of average fMRI response profiles using only a randomly selected half of the data.** The main finding - that the whisker-mediated tactile system clusters separately in awake but not in anesthetized animals - is similar to the Figure 5, where full dataset was used. Average fMRI response profiles were analyzed using hierarchical clustering. Correlation matrices on the left depict the similarity between frequency response profiles across regions-of-interest. The resulting hierarchical trees (dendrograms) are shown to the right of the matrices. Average response curves were subsequently calculated for four to five main clusters per group and are displayed on the far right. Clusters containing only a single region and high level of hierarchy were excluded from the illustrations. Regions without significant signal changes (Table 1) were also omitted from the analysis. The cluster including brainstem nuclei is color-coded in light blue across all groups. Abbreviations for regions-of-interest are provided in Figure 1 and in Table 1. Iso+Med, isoflurane and medetomidine anesthesia.


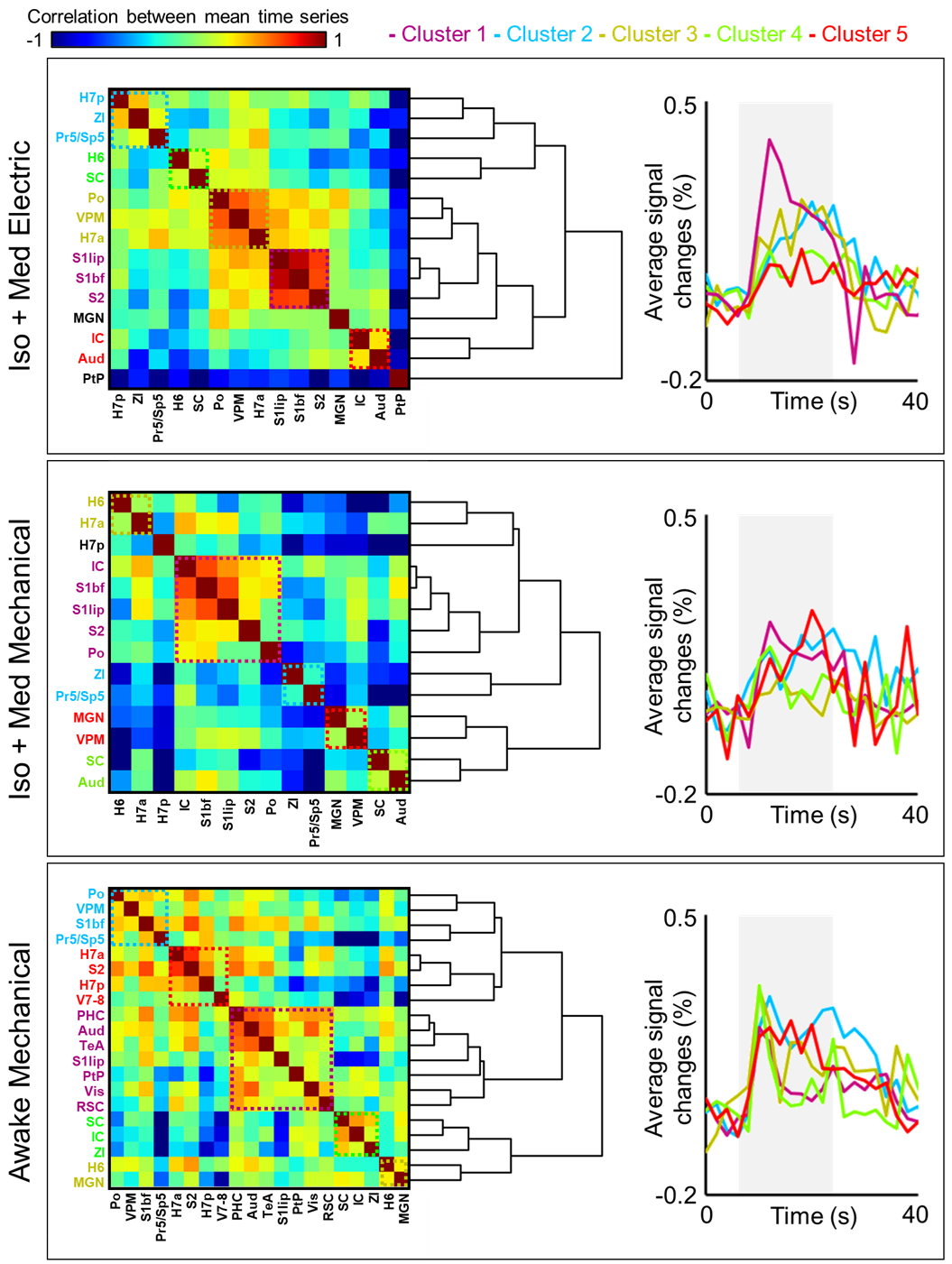


**Supplementary Figure 18. Hierarchical clustering of average fMRI time series using only a randomly selected half of the data.** The main finding - that the whisker-mediated tactile system clusters separately in awake but not in anesthetized animals – remains consistent to the Figure 6, where the full dataset was used. However, the lower correlation values observed here suggest a significant increase in noise levels. Average fMRI time series were analyzed using hierarchical clustering. Correlation matrices on the left indicate the similarity between time series across regions-of-interest. The resulting hierarchical trees (dendrograms) are shown to the right of the matrices. Subsequently, average time series were computed for five main clusters per group and are displayed on the far right. Clusters containing only a single region and high level of hierarchy were excluded from the illustrations. Regions without significant signal changes (Table 1) were also omitted from the analysis. The cluster including brainstem nuclei is color-coded in light blue across all groups. Abbreviations for regions-of-interest are provided in Figure 1 and in Table 1. Iso+Med, isoflurane and medetomidine anesthesia.
